# Decoding Macrophage Subtypes to Engineer Modulating Hydrogels for the Alleviation of Intervertebral Disk Degeneration

**DOI:** 10.1002/advs.202304480

**Published:** 2023-11-08

**Authors:** Da‐Wang Zhao, Qian Cheng, Huimin Geng, Jinbo Liu, Yuanqiang Zhang, Jiwei Cui, Chao Liu, Lei Cheng

**Affiliations:** ^1^ Department of Orthopedics Qilu Hospital of Shandong University Jinan Shandong 250012 China; ^2^ Key Laboratory of Colloid and Interface Chemistry of the Ministry of Education School of Chemistry and Chemical Engineering Shandong University Jinan Shandong 250100 China; ^3^ Department of Oral and Maxillofacial Surgery Qilu Hospital of Shandong University Jinan Shandong 250012 China; ^4^ Department of Oral Surgery, Shanghai Key Laboratory of Stomatology National Clinical Research Center of Stomatology Ninth People's Hospital Shanghai Jiao Tong University School of Medicine Shanghai 200011 China

**Keywords:** hydroxyapatite nanorods, intervertebral disk degeneration, macrophage polarization, polymer hydrogels, polyphenol

## Abstract

A major pathological basis for low back pain is intervertebral disk degeneration, which is primarily caused by the degeneration of nucleus pulposus cells due to imbalances in extracellular matrix (ECM) anabolism and catabolism. The phenotype of macrophages in the local immune microenvironment greatly influences the balance of ECM metabolism. Therefore, the control over the macrophage phenotype of the ECM is promising to repair intervertebral disk degeneration. Herein, the preparation of an injectable nanocomposite hydrogel is reported by embedding epigallocatechin‐3‐gallate‐coated hydroxyapatite nanorods in O‐carboxymethyl chitosan cross‐linked with aldehyde hyaluronic acid that is capable of modulating the phenotype of macrophages. The bioactive components play a primary role in repairing the nucleus pulposus, where the hydroxyapatite nanorods can promote anabolism in the ECM through the nucleopulpogenic differentiation of mesenchymal stem cells. In addition, epigallocatechin‐3‐gallate can decrease catabolism in the ECM in nucleus pulposus by inducing M2 macrophage polarization, which exists in normal intervertebral disks and can alleviate degeneration. The nanocomposite hydrogel system shows promise for the minimally invasive and effective treatment of intervertebral disk degeneration by controlling anabolism and catabolism in the ECM and inhibiting the IL17 signaling pathway (M1‐related pathway) in vitro and in vivo.

## Graphical abstract

An injectable nanocomposite hydrogel embedded with epigallocatechin‐3‐gallate‐coated hydroxyapatite nanorods is reported for the alleviation of intervertebral disk degeneration. This work highlights that the nanocomposite hydrogel is able to promote anabolism and decrease catabolism in the extracellular matrix (ECM) in nucleus pulposus by inducing M2 macrophage polarization, which exists in normal intervertebral disks and can alleviate degeneration of IVD.

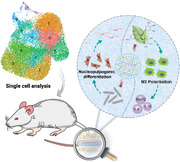

## 1 Introduction

Intervertebral disk (IVD) degeneration (IVDD) is a major pathological basis for low back pain.^[^ ^1^, ^2^ ^]^ IVDs are composed of cartilaginous endplates, external annulus fibrosus (AF), and nucleus pulposus (NP), where the NP is highly hydrated and capable of maintaining IVD height and uniform load transfer throughout the cartilaginous endplates.^[^ ^3^ ^]^ The degeneration of NP is a cascade caused by the imbalance between extracellular matrix (ECM) anabolism and catabolism, where anabolism is decreased in degenerated NP cells (NPC), while catabolism is promoted by the increased expression of matrix metalloproteinases (MMPs).^[^ ^4^ ^]^ The intricate interplay between ECM anabolism and catabolism holds significant relevance within the context of the immune microenvironment.^[^ ^5^ ^]^ In the milieu of human IVDD tissue, a confluence of granulation tissue, neovascularization, and CD68^+^ macrophages are evident. This phenomenon is concomitant with the upregulation of pro‐inflammatory factors, notably IL1β and TNFα.^[^ ^6^ ^]^ Any inflammatory response‐related cytokines cause altered mechanical function and ECM catabolism.^[^ ^7^, ^8^ ^]^ The underlying mechanism is that macrophage phenotype 1 (M1) increased the production of proteolytic enzymes on ECM, such as MMP (e.g., MMP1, MMP3, MMP9, and MMP13) and aggrecanases (ADAMTS), which contributed to ECM catabolism.^[^ ^9^, ^10^ ^]^ Moreover, a large number of macrophages have been observed to infiltrate around the prominent NP tissue,^[^ ^11^ ^]^ however the relative quantity and evolutionary ratio of M1 and macrophage phenotype 2 (M2) cells in IVDD has not been studied, nor has the modulation of this population been explored.

A key feature of IVDs is that they are not vascularized, and therefore systemic administration is not useful for treating degeneration.^[^ ^12^ ^]^ In addition, IVDs are surrounded by ligaments and muscles, which makes it a shock absorbing system that for withstanding forces and energy from any direction. For the past few years, injectable hydrogels have attracted attention for IVDD because of their minimally invasive nature, ability to conformally fill damaged areas, and control the loading and release of therapeutics. While a number of biodegradable polymers such as proteins, polyesters, and polyphosphazenes have been used for the preparation of injectable hydrogels,^[^ ^13^ ^]^ biocompatible and degradable hydrogels based on polysaccharides are regarded as superior carriers to stabilize, deliver, and slowly release of nanoparticles at the NP on account of their favorable biosafety, easy preparation, and similarity to ECM.^[^ ^14^ ^]^ Still, ideal therapeutics and formulations for IVDD are rare, despite the recent progress in hydrogels.

Hydroxyapatite (HAP) is a naturally occurring mineral in many organisms,^[^ ^15^ ^]^ and therefore HAP nanostructures have exceptional biocompatibility, appropriate biodegradability, and can promote chondrocyte hypertrophy and differentiation,^[^ ^16^, ^17^ ^]^ which play a crucial role in promoting the production (anabolism) of ECM (collagen type II [COL2] and glycosaminoglycan [GAG]).^[^ ^18^ ^]^ Epigallocatechin‐3‐gallate (EGCG) is a plant polyphenol with high biological activity and low toxicity that can regulate the immune microenvironment by promoting M2 polarization and reduce ECM catabolism, and also adhere firmly to various substrates via multiple anchoring interactions under mild conditions to spontaneously form coatings.^[^ ^19^, ^20^, ^21^ ^]^ Therefore, we hypothesized that ECGC‐coated HAP nanorods could promote NP regeneration in IVDD by inducing ECM anabolism and inhibiting ECM catabolism, where a polysaccharide‐based hydrogel could act as the ideal delivery system.

**Scheme 1:**
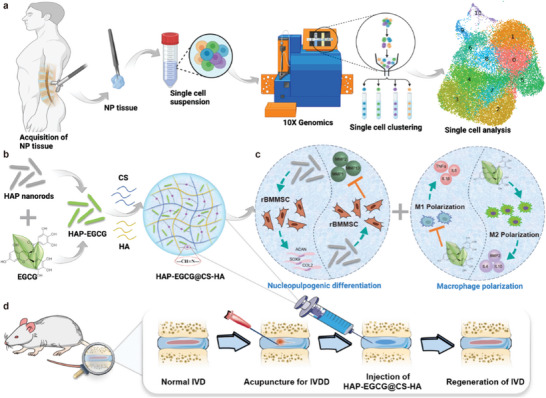
Schematic diagram of the experimental procedure. a) Schematic workflow of the single‐cell RNA‐sequence experiment. b) Preparation of HAP‐EGCG@CS‐HA hydrogels. c) rBMMSCs seeded on hydrogels in vitro, where HAP‐EGCG@CS‐HA hydrogels can induce ECM anabolism (*ACAN*, *SOX9*, and *COL2*) and downregulate the expression of MMPs. Macrophages were seeded on HAP‐EGCG@CS‐HA hydrogels in vitro to induce M2 polarization and anti‐inflammatory cytokines (IL4 and IL10). d) Schematic representation of IVD regeneration mediated by HAP‐EGCG@CS‐HA hydrogels in the in vivo IVDD model.

The construction of functional materials with nanoscale components through nanoarchitectonics is of great inspiration for the development of biotechnology.^[^ ^22^, ^23^, ^24^, ^25^ ^]^ In this work, an injectable and biodegradable nanocomposite hydrogel was prepared to orchestrate macrophage polarization and nucleopulpogenic differentiation according to the single‐cell atlas of human NP (**Scheme** **1**). First, HAP‐EGCG nanorods were obtained by the oxidative polymerization of EGCG on the surface of HAP nanorods. Next, the coated nanorods were loaded into an injectable hydrogel formed via the Schiff base reaction of O‐carboxymethyl chitosan (CS) and aldehyde hyaluronic acid (HA) to produce HAP‐EGCG@CS‐HA hydrogels. Rat bone marrow mesenchymal stem cells (rBMMSCs) and macrophages were cultured with HAP‐EGCG@CS‐HA hydrogels in three culture modes (leaching liquor, on the surface of hydrogels, and conditioned culture medium). We observed the biocompatibility of the nanocomposite hydrogel (89.2% cell viability), the nucleopulpogenic differentiation of rBMMSCs (over twofolds of ACAN, SOX9, and COL2 expression), and M2 polarization (23.0% VS 6.4%) in vitro. We further evaluated the regulatory effects of HAP‐EGCG@CS‐HA hydrogels on ECM anabolism and catabolism in vivo, and saw improved gray value of IVD imaging at 4 and 8 weeks, which are expected to play a role in the treatment of IVDD.

## 2 Results and Discussion

### 2.1 Single‐Cell RNA‐Sequencing and Clustering of Macrophages in Human NP Tissue

**Figure 1:**
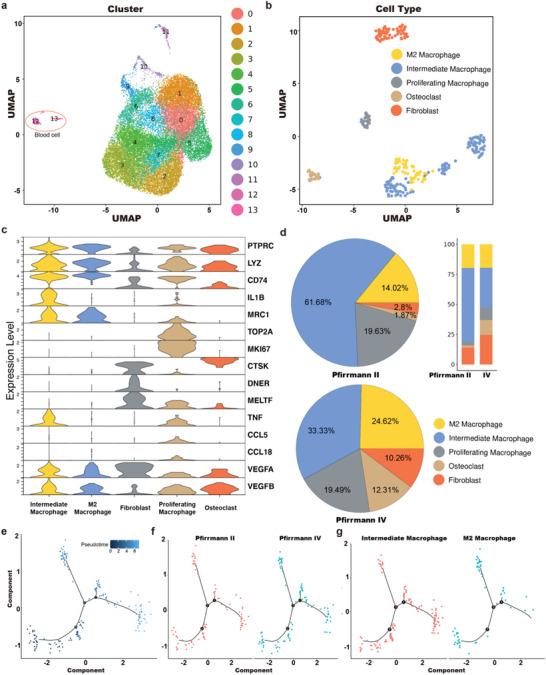
A single‐cell atlas of NP from humans with IVDD. a) UMAP view of 26882 single cells, color‐coded by assigned cell cluster. b) UMAP view of NP blood cells, color‐coded by subtypes. c) Violin plot map of typical expressed genes in each cell subtype. d) Pie charts and bar plot depicting the proportions of cell subtypes within different gradings of NP tissue. e) Monocle pseudotime analysis showing the progression of the macrophages. f) Monocle pseudotime analysis showing the progression of the macrophages in Pfirmann II than in Pfirmann IV patients. g) Monocle pseudotime analysis showing the progression of M2 and intermediate macrophages.

To determine the changes in macrophage clustering and molecular biological information in NP cells from humans with IVDD, we compared NP tissue isolated from Pfirrmann II and IV patients through Single‐cell RNA‐seq (**Figure** **1**a). There were 26 882 cells isolated from NP tissue and they were divided into 13 clusters using surface markers, with clusters 12 and 13 defined as blood cells.^[^ ^26^ ^]^ Based on the visualized results of marker genes and the relative positions using Uniform Manifold Approximation and Projection (UMAP), blood cells were divided into several subgroups, including fibroblasts, M2 cells, intermediate macrophages, osteoclasts, and proliferating macrophages (Figure 1b).^[^ ^27^ ^]^ The representative gene markers are shown in the violin diagram in Figure 1c. The expressions of M2 cell related genes (MRC1, CCL18, VEGFA, and VEGFB) and M1 cell related genes (IL1β) were both upregulated in intermediate macrophages.^[^ ^28^, ^29^ ^]^ The number of M2 cells in Pfirrmann II and IV patients was similar, while the number of fibroblasts in Pfirrmann IV patients was significantly higher than that in Pfirrmann II, indicating severe fibrosis in the NP tissue of Pfirrmann IV patients (Figure 1d). More intermediate macrophages were seen in Pfirmann II patients, and the results indicated that more M1 cells would transform into M2 cells in low‐grade IVDD (Pfirrmann II), which is beneficial for tissue repair. Further pseudotime analysis of macrophage subtypes revealed the early, middle, and late stages of cell differentiation (Figure 1e, located at the ends of curves 2, 3, and 1, respectively), where most of the cells in Pfirmann IV patients are in the late stage of differentiation, which indicated a decrease in cell function (Figure 1f). Most of the intermediate macrophages were also in the late stage of differentiation, which indicated that they were close to M2 cells (Figure 1g). Therefore, it is reasonable to infer that there are more intermediate macrophages (soon to be M2) in healthy IVD than in degenerating IVD,^[^ ^30^ ^]^ which provides a new insight for regulating macrophage subtypes and treating IVDD.

### 2.2 Preparation and Characterization of HAP‐EGCG@CS‐HA Hydrogels

An injectable nanocomposite hydrogel was prepared by mixing solutions of CS and HA loaded with HAP‐EGCG nanorods (HAP‐EGCG@CS‐HA hydrogels). First, the structures of CS and HA were characterized by ^1^H nuclear magnetic resonance (^1^H‐NMR) (Figure S1, Supporting Information), where the peak at 2.7 ppm in the ^1^H‐NMR spectrum of CS corresponds to the carboxymethyl group grafted onto the hydroxyl groups. HA prepared by the reaction of sodium hyaluronate with sodium periodate has two peaks at 4.9 and 5.0 ppm in its ^1^H‐NMR spectrum, corresponding to hemiacetalic protons formed from the aldehyde groups and neighboring hydroxyl groups.

**Figure 2:**
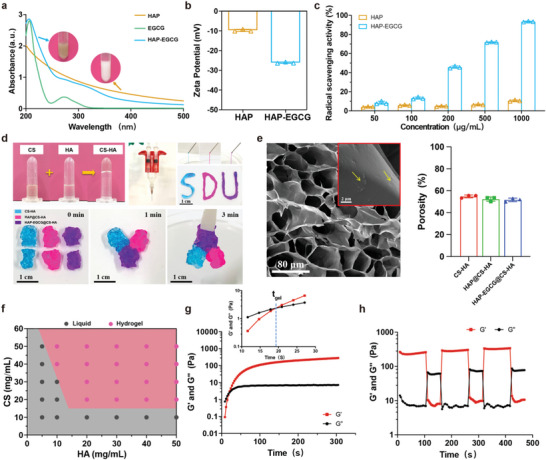
Preparation and characterization of HAP‐EGCG@CS‐HA hydrogels. a) UV–vis spectra of HAP nanorods, EGCG, and HAP‐EGCG nanorods; the inset photographs are solutions of HAP nanorods and HAP‐EGCG, respectively. b) The zeta potential of HAP nanorods and HAP‐EGCG nanorods. c) DPPH scavenging properties of HAP and HAP‐EGCG at different concentrations. d) Photographs of the hydrogels prepared by mixing CS and HA solution (blue is CS‐HA, red is HAP@CS‐HA, and purple is HAP‐EGCG@CS‐HA) and the process used to demonstrate injectability and self‐healing. e) SEM images of HAP‐EGCG@CS‐HA hydrogels (the arrows point to HAP‐EGCG nanorods), and the porosity percentage of the hydrogels. f) Phase diagrams of mixtures of CS and HA. g) Storage modulus (G′) and loss modulus (G′′) of HAP‐EGCG@CS‐HA hydrogels over time. The concentrations of CS and HA were 40 and 30 mg mL^−1^, respectively, with 200 µg mL^−1^ HAP‐EGCG nanorods. The crossover time point of the G′ and G′′ curves is defined as the mechanical gelation point (*t*_gel_). h) Measurement of G′ and G′′ over time in a step sweep test conducted by applying a low strain (γ = 0.1%) to the hydrogel for the first 100 s, then a high strain (γ = 1000%) for the next 50 s, which was repeated twice.

HAP‐EGCG nanorods were obtained by mixing EGCG and HAP nanorods in an alkaline solution (pH 8.5), which led to a color shift from white to brown (**Figure** **2**a). The UV–vis absorption peak of EGCG at 206 nm was identifiable in dispersions of HAP‐EGCG, suggesting successful coating.^[^ ^31^ ^]^ Transmission electron microscopy (TEM) images confirmed that coating did not change the morphology of the nanorods (Figure S5, Supporting Information) but did decrease the zeta‐potential of the nanorods (Figure 2b). Compared with the HAP nanorods, the FTIR spectra of HAP‐EGCG showed three characteristic peaks representing aromatic hydrocarbons at 1650—1430 cm^−1^ (Figure S2, Supporting Information), confirming that there was an EGCG coating on the surface of the HAP nanorods. Furthermore, as a natural polyphenol, EGCG is an excellent antioxidant and showed strong free radical scavenging ability. Specifically, HAP‐EGCG nanorods could scavenge 50% of 1,1‐diphenyl‐2‐picrylhydrazyl (DPPH) at a concentration of 200 µg mL^−1^ (Figure 2c), indicating the potential to inhibit the inflammatory responses. The 200 µg mL^−1^ HAP‐EGCG induced M2 polarization, providing supportive results for our subsequent experiments (Figure S3, Supporting Information).

CS and HA were dissolved in water, and after mixing, a hydrogel (CS‐HA) formed immediately (Figure 2d), avoiding the use of chemical linkers or light. In the Fourier‐transform infrared (FTIR) spectra (Figure S4, Supporting Information), a characteristic peak at 1639 cm^−1^ was observed in the spectrum of the hydrogels, which was assigned to the characteristic ν_C = N_ stretching vibration.^[^ ^32^ ^]^ These results indicated that the Schiff base bonds between the amino groups of CS and the aldehyde groups of HA contributed to hydrogel formation. The dynamic Schiff base bonds make CS‐HA hydrogels injectable and potentially self‐healing. For example, CS‐HA hydrogels loaded with HAP nanorods or HAP‐EGCG nanorods could be injected through a narrow needle (26 G) after mixing equivalent volumes of CS and HA solutions containing HAP nanorods in separate syringes with a Y‐shaped connector. When put into contact, pieces of different hydrogels (i.e., CS‐HA, HAP@CS‐HA, and HAP‐EGCG@CS‐HA) could gradually merge into a whole hydrogel within 3 min, demonstrating the self‐healing properties of the hydrogels. Meanwhile, the degradable properties of the hydrogels were demonstrated in vitro (Figure S6, Supporting Information), where 50% of the hydrogels were degraded after one week, and only 20% of the original mass remained after two weeks. Scanning electron microscopy (SEM) images demonstrated that HAP‐EGCG@CS‐HA hydrogels had a porous structure, and HAP‐EGCG nanorods were uniformly distributed without damaging the microstructures of CS‐HA hydrogels (Figure 2e). Furthermore, the porosity of hydrogels was measured by ethanol displacement method, and the result showed that the percentage of porosity for all hydrogels (CS‐HA, HAP@CS‐HA, and HAP‐EGCG@CS‐HA) was greater than 50%, which provides favorable conditions for cell survival and migration.

The concentrations of CS and HA could be adjusted over a wide range and still form hydrogels due to the strong and rapid Schiff base reaction between amino groups and aldehyde groups. Specifically, hydrogel formation required a CS concentration of 20 mg mL^−1^ or higher and an HA concentration 10 mg mL^−1^ or higher (Figure 2f). Rheological analysis revealed that the change in storage modulus (G') and loss modulus (G″) reflected the transition state of the hydrogel, where G′ was smaller than G″ at the beginning, corresponding to the sol state of the system (Figure 2g). Subsequently, G′ increased more rapidly than G″, leading to gelation (*t*_gel_) at 18 s due to the formation of Schiff base bonds between CS and HA. When the time was larger than *t*_gel_, G′ was significantly larger than G″, which implied the formation of a stable hydrogel. A step sweep test was conducted by applying a low strain (γ = 0.1%) to the hydrogel for the first 100 s and then a high strain (γ = 1000%) for the next 50 s, and repeated twice. The gels were unaffected (G′> G″) at a low strain and became a sol (G′ < G″) under a high strain (Figure 2h). This result confirmed the self‐healing ability of the hydrogels, which benefits from dynamic Schiff base bonds.

### 2.3 In Vitro Biocompatibility of HAP‐EGCG@CS‐HA Hydrogels

After alcohol gradient dehydration, the cellular adhesion and morphology of different hydrogels were analyzed using SEM. Due to the non‐cell‐adhesive properties of the polysaccharide hydrogels, rBMMSCs had a spherical morphology on the hydrogels as they maintained the chondrocyte phenotype (Figure S7, Supporting Information).^[^ ^33^ ^]^ The size of the rBMMSCs on the hydrogels was ≈8 µm in diameter (8.1 ± 0.8 µm on CS‐HA hydrogels, 8.2 ± 0.5 µm on HAP@CS‐HA hydrogels and 8.1 ± 0.4 µm on HAP‐EGCG@CS‐HA hydrogels), and macrophages were smaller than the rBMMSCs (5.4 ± 0.5 µm on CS‐HA hydrogels, 5.3 ± 0.4 µm on HAP@CS‐HA hydrogels and 5.3 ± 0.3 µm on HAP‐EGCG@CS‐HA hydrogels). Cell percent viability was calculated at 0, 2, 4, 6, and 8 days using a Methyl Thiazolyl Tetrazolium (MTT) test, and no significant difference in either rBMMSCs or macrophages can be seen over time (Figure S8, Supporting Information). Moreover, the addition of HAP and EGCG did not affect the proliferation of the two cell types studied, and images of a live/dead cell staining experiment (width, 1200 µm; height, 1200 µm; depth, 600 µm) randomly captured by a High Content Screening System also demonstrated that the hydrogels had negligible impact on cell viability (Figure S9, Supporting Information). Specifically, the ratio of live (green)/dead (red) cells (56.6 ± 4.9 on CS‐HA hydrogels, 57.6 ± 22.3 on HAP@CS‐HA hydrogels and 62.2 ± 13.8 on HAP‐EGCG@CS‐HA hydrogels for rBMMSCs; 63.3 ± 17.2 on CS‐HA hydrogels, 59.2 ± 16.7 on HAP@CS‐HA hydrogels and 62.0 ± 14.0 on HAP‐EGCG@CS‐HA hydrogels for macrophages) were not significantly different. The average live/dead cell ratio among all groups was above 56.6, indicating that the hydrogel coculture systems did not have a significant effect on the survival of either rBMMSCs or macrophages. The live/dead staining results confirm that the polysaccharide‐based hydrogels are ideal scaffolds for tissue engineering because of their similarity to the ECM, which is comprised of various amino acids and sugar‐based macromolecules.^[^ ^34^ ^]^ Additionally, the 3D hydrogels are highly porous, where the pores can support the growth and proliferation of cells encapsulated in the hydrogels.

To understand the apoptosis status of the entire system more comprehensively, cellular apoptosis was detected by Annexin V/7‐AAD staining on day 1. The proportion of rBMMSCs at late apoptosis was less than or equal to 1.1% (CS‐HA hydrogels, 0.8%; HAP@CS‐HA hydrogels, 1.1%; and HAP‐EGCG@CS‐HA hydrogels, 1.0%), and there was no difference between the groups (Figure S10, Supporting Information). The proportion of macrophages at early or late apoptosis was slightly higher, but the proportions were all less than or equal to 9.9%, and there was no difference between the groups. According to the above results, we found that HAP‐EGCG@CS‐HA hydrogels had high biological safety and showed good biocompatibility, confirming their promise for in vivo application.

### 2.4 Nucleopulpogenic Differentiation of rBMMSCs

It was previously reported that degeneration of IVDs causes a shift from COL2 to COL1 expression, overexpression of MMPs, and a decrease in ACAN anabolism, which leads to lower mechanical properties of dehydrated NPC.^[^ ^35^, ^36^ ^]^ The ECM anabolism and catabolism at the mRNA level were detected by measuring expression‐related genes, including *ACAN*, *SOX9*, *COL2*, *MMP2*, and *MMP13*. Compared with CS‐HA hydrogels, HAP@CS‐HA hydrogels led to an increase in *ACAN*, *SOX9*, and *COL2* mRNA levels, but there was no significant difference between HAP@CS‐HA and HAP‐EGCG@CS‐HA hydrogels, indicating that ECM anabolism of rBMMSCs was mainly due to the HAP nanorods (Figure S11, Supporting Information). The HAP nanorods had a better effect than these of granular structures in stimulating cell function, and they were more easily internalized than nanowires structures.^[^ ^37^ ^]^ HAP can release Ca^2+^ slowly,^[^ ^38^ ^]^ and the Ca^2+^ released by HAP nanorods gathered and flowed into rBMMSCs through the activated calcium‐sensing receptor (CaSR). Elevated extracellular calcium concentrations and actived CaSR can stimulate the differentiation of growth plate chondrocytes,^[^ ^39^ ^]^ thus HAP nanorods enhance the nucleopulpogenic differentiation of stem cells through a calcium ion‐dependent signaling pathway. In addition, lower *MMP2* and *MMP13* mRNA expressions were shown in the HAP‐EGCG@CS‐HA group (Figure S11, Supporting Information), which indicated that the EGCG coating could downregulate MMP expression. The ACAN expression of rBMMSCs was much higher in HAP‐EGCG@CS‐HA hydrogels than in the other systems (Figures S12 and S13, Supporting Information). In contrast, the lowest MMP2 expression was found in HAP‐EGCG@CS‐HA hydrogels, followed by HAP@CS‐HA and CS‐HA hydrogels. The EGCG coating upregulated the expression of ACAN and downregulated the expression of MMP2. Collectively, these results indicated that HAP‐EGCG@CS‐HA hydrogels could promote ECM anabolism and inhibit ECM catabolism.

### 2.5 Evaluation of Macrophage Polarization

**Figure 3:**
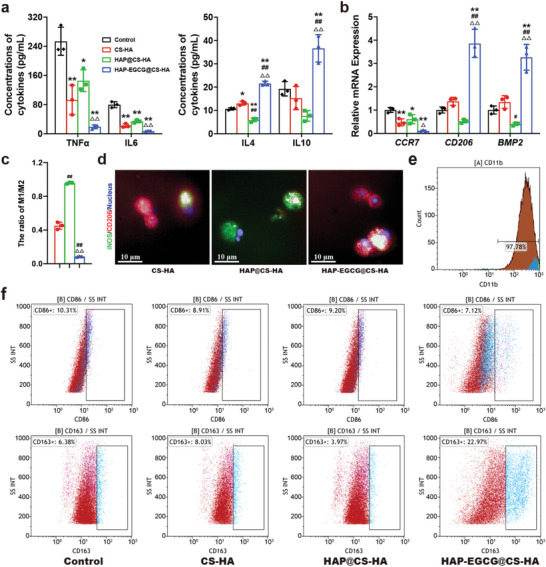
HAP‐EGCG@CS‐HA hydrogels regulates macrophage polarization. a) TNFɑ, IL6, IL4, and IL10 production by macrophages cultured in leaching liquors of the hydrogels on day 3 by ELISA. b) *CCR7, CD206*, and *BMP2* mRNA expression of macrophages cultured in leaching liquors of the hydrogels on day 3 by RT‐PCR. c) Quantitative analysis of IF staining for surface markers (iNOS and CD206) of macrophages cultured on hydrogels for 3 days. d) Images of macrophages cultured on hydrogels for 3 days with the surface markers (iNOS and CD206) labeled by IF staining. e) Identification of macrophage surfaces marker by flow cytometry, demonstrating that the cells were positive for CD11b. f) Flow cytometry analysis of the M1 phenotype marker CD86 and the M2 phenotype marker CD163. The analysis was performed on macrophages cultured in leaching liquors of the hydrogels on day 3. (n = 3. The values presented are the mean±SD. Statistical significance was assessed by one‐way ANOVA with a post‐hoc bonferroni test. *, #, and △ represent *P* <0.05 compared with control, CS‐HA hydrogels, and HAP@CS‐HA hydrogels, respectively; **, ##, and △△ represent *P* <0.01 compared with control, CS‐HA hydrogels, and HAP@CS‐HA hydrogels, respectively)

It has been shown that M2 cells promote proliferation and the ECM synthesis of NPC, while inhibiting inflammatory responses, NPC apoptosis, and senescence.^[^ ^40^ ^]^ Macrophages (6×10^4^ mL^−1^) were cultured in leaching liquor or on hydrogels for 3 days, the HAP‐EGCG@CS‐HA hydrogels significantly inhibited production of the proinflammatory cytokines (TNFα and IL6) according to the results of ELISA (**Figure** **3**a; Figure S14, Supporting Information), which indicated that HAP‐EGCG@CS‐HA hydrogels can reduce the secretion of proinflammatory cytokines. In contrast, secretion of IL4 and IL10 by M2 macrophages was significantly upregulated by HAP‐EGCG@CS‐HA hydrogels.

RT‐PCR was carried out to detect the expression of M1 and M2‐related markers. Expression of *CCR7* (an M1 surface marker) was significantly decreased in the presence of HAP‐EGCG@CS‐HA hydrogels but enhanced in the control group (Figure 3b). In contrast, there was a clear expression enhancement of the M2 surface marker *CD206* in the HAP‐EGCG@CS‐HA hydrogels sample. Moreover, macrophages grown in HAP‐EGCG@CS‐HA hydrogels had significantly higher *BMP2* expression than macrophages in the control group (Figure 3b). HAP‐EGCG@CS‐HA hydrogels featured an obviously lower percentage of iNOS‐positive cells than the other groups (Figure 3c,d), however, more CD206 expression was detected in the HAP‐EGCG@CS‐HA group. To accurately quantify the ratio of M1 to M2 macrophages, the number of each type of cell was calculated using the High Content Screening System. On the third day of culture, there were more M2 cells and fewer M1 cells in the HAP‐EGCG@CS‐HA group than in the other groups (Figure 3c).

We performed flow cytometry to analyze the expression of the surface markers CD86 (M1 surface marker) and CD163 (M2 surface marker) simultaneously, and we found that >97% of the cells were positive for CD11b (Figure 3e). The percentage of CD11b^+^CD86^+^ cells was 7.1% in the HAP‐EGCG@CS‐HA group, which was 3.2% lower than that in the control group (Figure 3f). Furthermore, the percentage of CD11b^+^CD163^+^ cells in the HAP‐EGCG@CS‐HA group (23.0%) was higher than those in the control (6.4%), CS‐HA hydrogels (8.0%), and HAP@CS‐HA hydrogels (4.0%) groups, which indicated that HAP‐EGCG@CS‐HA hydrogels tended to induce the transition from M1 to M2. Collectively, these results show that the addition of EGCG can induce M2 polarization, with the production of anti‐inflammatory cytokines (IL4 and IL10), and reduce the release of proinflammatory cytokines (TNFα and IL6). M2 macrophages inhibit TNFα‐induced IVDD effects, and therefore M2 promotes NPC proliferation and ECM synthesis and inhibits inflammatory responses and apoptosis of NPC. This complements previous studies that have shown that EGCG inhibits HIF1α signaling to block the proinflammatory phenotype in macrophages (M1),^[^ ^41^ ^]^ and inhibits the production of proinflammatory cytokines by reducing the expression of phosphorylated NF‐κB p65 to inhibit the activation of NF‐κB.^[^ ^41^ ^]^

### 2.6 Conditioned Medium Culture System

**Figure 4:**
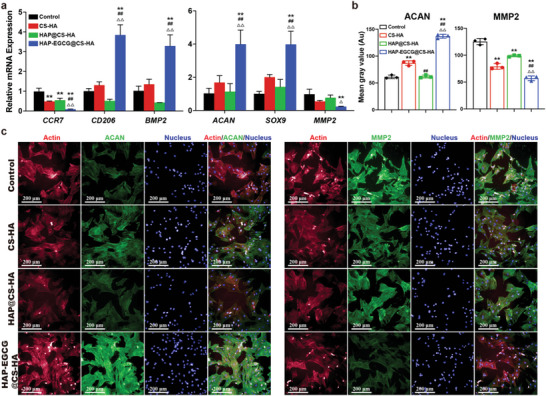
HAP‐EGCG@CS‐HA hydrogels regulates the nucleopulpogenic differentiation of rBMMSCs and the polarization of macrophages in a conditioned medium culture system. a) Expression of *CCR7*, *CD206*, and *BMP2* (macrophages) and *ACAN*, *SOX9*, and *MMP2* (rBMMSCs) in the conditioned medium culture system on day 2 by RT‐PCR. b) Quantitative analysis of rBMMSCs with ACAN or MMP2. c) rBMMSCs with ACAN or MMP2 in the conditioned medium culture system on day 2 by IF staining. (n = 3. The values presented are the mean±SD. Statistical significance was assessed by one‐way ANOVA with a post‐hoc bonferroni test. *, #, and △ represent *P* <0.05 compared with control, CS‐HA hydrogels, and HAP@CS‐HA hydrogels, respectively; **, ##, and △△ represent *P* <0.01 compared with control, CS‐HA hydrogels, and HAP@CS‐HA hydrogels, respectively)

To further study the effect of hydrogel‐induced macrophage polarization on ECM anabolism and catabolism in vitro, rBMMSCs were cultured in a macrophage‐conditioned culture medium as described in the methods section. The expression of macrophage‐related genes (*CD206* and *BMP2*) demonstrated that HAP‐EGCG@CS‐HA hydrogels tended to induce M2 polarization (**Figure** **4**a, left panel). Moreover, the expression of *ACAN* and *SOX9* was significantly higher in HAP‐EGCG@CS‐HA hydrogels than in the other groups, but the expression of *MMP2* was the lowest (Figure 4a, right panel). We used IF staining and quantitative analysis to detect ACAN and MMP2 on day 2 of culturing, and the expression of ACAN in the HAP‐EGCG@CS‐HA group was more than twice that in the HAP@CS‐HA group (Figure 4b,c). In contrast, the lowest MMP2 expression was found in the HAP‐EGCG@CS‐HA group, followed by the groups of CS‐HA and HAP@CS‐HA hydrogels. The cells spread better and had more pseudopodia in the HAP‐EGCG@CS‐HA group, which was different from the original cell morphology because the cells were cultured on the surface of the petri dish rather than in the hydrogel. The above results indicated that HAP‐EGCG@CS‐HA hydrogels could promote ECM anabolism and inhibit ECM catabolism.

IVDD is attributed to a complex interplay between ECM anabolism/catabolism and the immune microenvironment.^[^ ^42^ ^]^ On the one hand, an important feature of NP degeneration is the increased degradation of ACAN and other aggregating proteoglycans, which leads to imbalances between ECM anabolism and catabolism.^[^ ^43^ ^]^ In addition, ECM catabolism products could trigger the aggregation of inflammatory mediators, in turn leading to a degenerative process through MMP production.^[^ ^44^ ^]^ On the other hand, during the development of IVDD, high mobility group box protein 1 is significantly increased and promotes the production of inflammatory cytokines such as TNFα, prostaglandin E2 (PGE2), IL6, and MMPs.^[^ ^45^ ^]^ Next, the inflammatory cascade (e.g., M1 polarization‐related cytokines TNFα and IL1β) contributes to the degenerative process of NP degeneration, by causing altered mechanical function and interrupting the balance of ECM anabolism and catabolism in the NP (downregulation of COL2 and ACAN anabolism),^[^ ^7^ ^]^ which contribute to IVDD.^[^ ^46^ ^]^ Our results verified the effects of macrophage‐conditioned medium (control, CS‐HA hydrogels, HAP@CS‐HA hydrogels, and HAP‐EGCG@CS‐HA hydrogels) on rBMMSCs. By eliminating the interference of cell–cell interactions, we demonstrated that HAP‐EGCG@CS‐HA hydrogels induced M2 polarization and the secretion of anti‐inflammatory cytokines (IL4 and IL10) as exogenous factors that cooperate with HAP to promote the expression of proteins related to ECM anabolism (ACAN and SOX9). Anti‐inflammatory cytokines (IL4 and IL10) can also significantly reduce MMP2 production in the HAP group and inhibit catabolism of the ECM.

### 2.7 In Vivo Gene and Protein Expression in an IVDD Model

**Figure 5:**
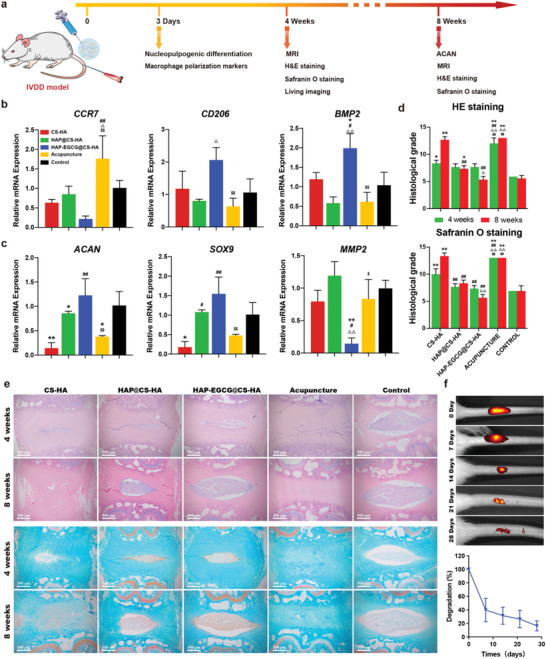
HAP‐EGCG@CS‐HA hydrogels in an IVDD model regulates macrophage polarization and ECM anabolism/catabolism. a) Flow chart for the in vivo evaluation of IVD regeneration. b) *CCR7*, *CD206*, and *BMP2* of IVD tissue 3 days after injection by RT‐PCR. c) *ACAN*, *SOX9*, and *MMP2* of IVD tissue 3 days after injection by RT‐PCR. d) Histological grade from HE and safranin O staining at 4 and 8 weeks after injection. e) HE and Safranin O staining of the IVD at 4 and 8 weeks after injection. f) Assessment of in vivo degradation rate of the IVD delivery system HAP‐EGCG@CS‐HA hydrogel. (n = 3. The values presented are the mean±SD. Statistical significance was assessed by one‐way ANOVA with a post‐hoc bonferroni test. *, #, △ and $ represent *P* <0.05 compared with control, CS‐HA hydrogels, HAP@CS‐HA hydrogels, and HAP‐EGCG@CS‐HA hydrogels, respectively; **, ##, △△ and $$ represent *P* <0.01 compared with control, CS‐HA hydrogels, HAP@CS‐HA hydrogels, and HAP‐EGCG@CS‐HA hydrogels, respectively)

The whole IVD was extracted 3 days after hydrogel injection and was subjected to mRNA extraction (**Figure** **5**a). RT‐PCR was carried out according to the manufacturer's instructions in vitro, where we found that the expression levels of the in vivo macrophage polarization‐related gene expression (*CCR7, CD206*, and *BMP2*) were similar to what was obtained in vitro (Figure 3b), showing that HAP‐EGCG@CS‐HA hydrogels could induce M2 polarization (Figure 5b). This demonstrated that HAP‐EGCG@CS‐HA hydrogels can cause changes to the immune microenvironment in the IVD. First, it induces M2 polarization, and then M2 produces anti‐inflammatory cytokines (IL4, IL10, etc.), thereby promoting ECM anabolism in vivo. The expression levels of the ECM anabolism‐related genes *ACAN* and *SOX9* in the IVD extracted from the HAP‐EGCG@CS‐HA group were significantly higher than those from the other groups, but there was no significant difference between the control and HAP@CS‐HA groups (Figure 5c). In contrast, the expression level of the ECM catabolism‐related gene *MMP2* in the HAP‐EGCG@CS‐HA group was significantly lower than that in the other groups (Figure 5c). This further indicated that HAP‐EGCG@CS‐HA hydrogels could create a better immunomodulatory environment for ECM anabolism.

Taken together, our results demonstrated that HAP‐EGCG@CS‐HA hydrogel injection protected the IVD from degeneration to some extent and even promoted tissue regeneration by modulating the immune microenvironment. This may be explained by the ability of M2 macrophages to adopt an anti‐inflammatory and tissue‐repairing phenotype that is important for promoting IVD repair. Next, we further explored the ability of the hydrogels to reconstruct IVD function and shape in vivo.

### 2.8 In Vivo MRI and Histology in an IVDD Model

MRI and histologic staining of sections were performed to assess the IVD regeneration grade in vivo, 4 and 8 weeks after injection, MRI reflects the water content of the IVD,^[^ ^47^ ^]^ and a higher grayscale value indicates a higher water content in T2‐weighted images. An MRI image was collected using untreated IVD to show the imaging features of normal IVD (week 0). The coronal images obtained for each group by MRI and the gray value analysis at 4 and 8 weeks demonstrated minimal changes in the control group, while the acupuncture group (needle, but no hydrogel injection) showed a significant reduction in gray values at 4 and 8 weeks (Figures S15 and S16, Supporting Information). The injection of CS‐HA hydrogels did not significantly improve the gray value changes caused by acupuncture, however HAP@CS‐HA and HAP‐EGCG@CS‐HA hydrogels improved the gray value changes caused by acupuncture at 4 and 8 weeks, and the effect was stronger than that of CS‐HA hydrogels. Therefore, based on MRI analysis, both HAP@CS‐HA and HAP‐EGCG@CS‐HA hydrogels alleviated the degeneration of the IVD.

Histologic sections (H&E and safranin O staining) were used to observe the histology of the NP and AF and the collagen expression of the IVD. We also calculated the histological score according to previous research.^[^ ^48^ ^]^ Eight weeks after injection, a clear border between NP and AF was shown in the HAP‐EGCG@CS‐HA group, as well as significant improvements in NP cellularity and morphology compared with that observed at 4 weeks (Figure 5d,e). Furthermore, in the CS‐HA and acupuncture groups, a blurred boundary between the annulus fibrosus and NP was observed, and the condition of the NP also deteriorated. Safranin O staining can be used to assess collagen levels in the IVD, and compared with the control groups, collagen levels were higher in the HAP‐EGCG@CS‐HA group and lower in the CS‐HA and acupuncture groups (Figure 5e). In the HAP‐EGCG@CS‐HA group, the histological grade was significantly lower at 8 weeks after injection, which indicated the regenerative effects of the hydrogel. Notably, the CS‐HA group had a higher histological grade at 8 weeks when compared to 4 weeks (Figure 5d), confirming the importance of the HAP‐EGCG nanorods. Dynamic monitoring of hydrogel degradation within the IVD has shown that degradation typically occurs over a period of ≈4 weeks (Figure 5f). During the initial stages, the hydrogel serves as a biological scaffold for IVD cells, releasing HAP‐EGCG to stimulate cell differentiation and regeneration. However, beyond the 2‐week mark, the degraded hydrogel scaffold begins to create space for the proliferation of new cells.

HAP can induce the ECM anabolism of rBMMSCs, but it cannot induce M2 polarization. The addition of EGCG can induce M2 polarization, increase the secretion of IL4 and IL10, and reduce the release of proinflammatory cytokines (TNFα and IL6). On the one hand, EGCG has been shown to promote M2 polarization, with increased expression of CD163 and CD206 (M2 markers), inhibiting the inflammatory response and downregulating the expression of MMPs, which decreases degradation of the ECM. On the other hand, EGCG has the ability to promote and enhance ECM anabolism, showing great potential in the modification of tissue engineering materials for the application of NP repair.^[^ ^49^ ^]^ Additionally, a single HAP cannot be delivered through injection. By loading EGCG‐coated HAP nanorods into the hydrogel, we created a HAP‐EGCG@CS‐HA hydrogels system in the IVD, which can effectively regulate ECM anabolism/catabolism and promote NP regeneration through M2 polarization.

### 2.9 Molecular Mechanism of Macrophage Polarization Analyzed by RNA Sequencing

**Figure 6:**
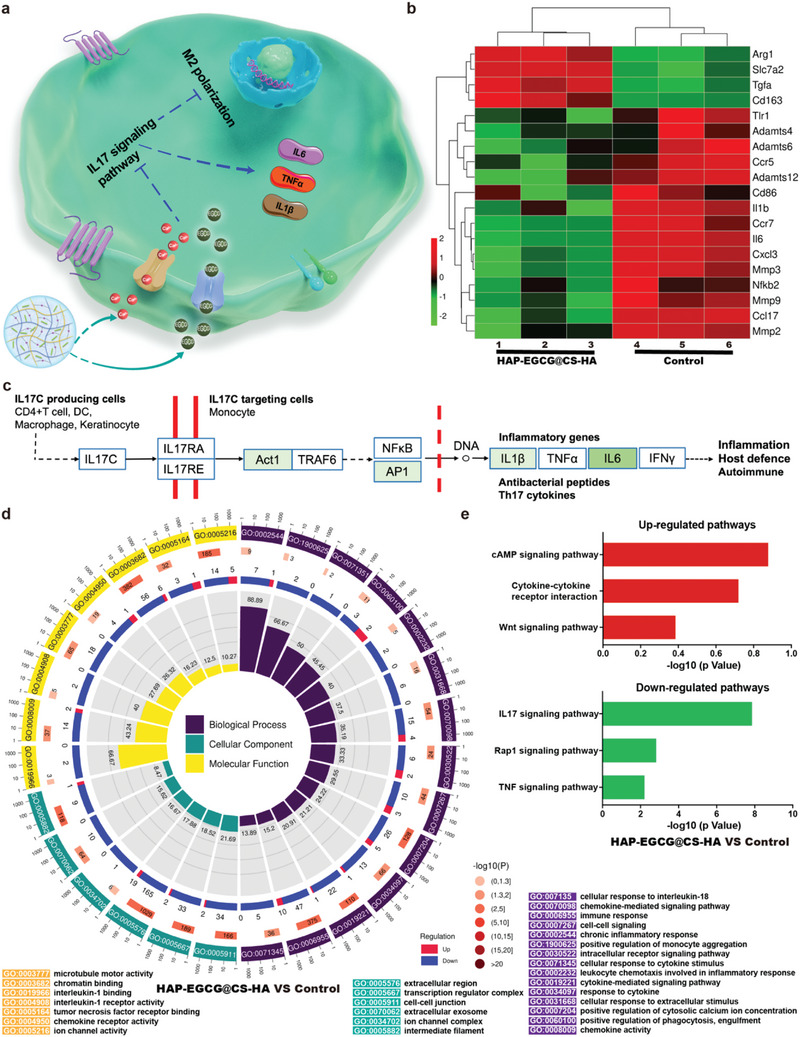
Molecular mechanism of macrophage polarization when cultured with HAP‐EGCG@CS‐HA hydrogels. a) Molecular mechanism of M2 polarization induced by HAP‐EGCG@CS‐HA hydrogels. b) The heat map of fold change in the expression of selected genes. c) Pathway analysis of the IL17 signaling pathway based on the KEGG database. The green boxes represent down‐regulated genes. d) GO analysis of all genes in macrophages cultured with the HAP‐EGCG@CS‐HA group versus the control group. e) Top three upregulated and top three downregulated pathways analyzed by the KEGG pathway method.

The regulation of immune response is an important part for ECM anabolism. Ca^2+^ (released by HAP) and EGCG (released by HAP‐EGCG) have been shown to be important for immune responses and promote M2 polarization.^[^ ^50^ ^]^ The HAP‐EGCG@CS‐HA hydrogels induced M2 polarization and regulated the production of anti‐inflammatory factors in our study. To understand the precise biomolecular mechanisms of macrophage polarization induced by HAP‐EGCG@CS‐HA hydrogels, RNA sequencing was performed to analyze signaling pathway differences in macrophages between the HAP‐EGCG@CS‐HA and control group (**Figure** **6**a). The expressions of M2‐related genes (*Arg1* and *CD163*) were clearly enhanced in the HAP‐EGCG@CS‐HA group along with the expression of *Slc7a2* (reconstructive process gene) (Figure 6b). The expression levels of an M1 marker (*CD86*) and related genes (*IL1β and IL6*) were clearly decreased in the HAP‐EGCG@CS‐HA group, while The ECM catabolism genes (*MMPs* and *ADAMTS*) also decreased in HAP‐EGCG@CS‐HA group, which is beneficial for ECM production. Three signaling pathways, including the cAMP, cytokine–cytokine receptor interaction, and Wnt signaling pathway, were up‐regulated, where the cAMP and Wnt signaling pathways are associated with the polarization of macrophages (Figure 6c,e).^[^ ^51^, ^52^ ^]^ The IL17 signaling pathway (M1‐related pathway) was significantly downregulated in the HAP‐EGCG@CS‐HA group,^[^ ^53^ ^]^ including down‐regulation of *ACT1*, *AP1*, *IL1β*, and *IL6* (Figure 6c). Next, the differentially expressed genes in the HAP‐EGCG@CS‐HA and control groups were collected and subjected to GO enrichment analysis (Figure 6d). The differential genes of cells are generally divided into three classical parts (biological process, molecular function, and cellular components). The 28 enrichment terms of the HAP‐EGCG@CS‐HA versus the control group demonstrated enrichment in the regulation of genes relating to cell–cell signaling, chemokine activity, and immune response, which were induced by HAP‐EGCG@CS‐HA hydrogels related M2 polarization through IL17 signaling pathway (Figure 6d).

## 3 Conclusion 

In conclusion, we found that the number of intermediate macrophages (soon to be M2) in degenerated IVD was less than that in normal IVD according to the results of single‐cell RNA‐seq. Then, we prepared an injectable and biodegradable nanocomposite hydrogel by cross‐linking CS and HA loaded with HAP‐EGCG nanorods (HAP‐EGCG@CS‐HA hydrogels) to achieve a better and longer‐lasting treatment for IVDD. The study revealed that macrophages induced molecular changes associated with IVDD. This included an increase in the expression of key matrix catabolic genes, as well as a reduction in the expression of major matrix‐associated anabolic genes.^[^ ^54^ ^]^ The simultaneous effects of the HAP‐EGCG nanorods effectively repaired NP through the promotion of ECM anabolism via the nucleopulpogenic differentiation of rBMMSCs and the inhibition of ECM catabolism due to M2 polarization. We demonstrated that HAP‐EGCG@CS‐HA hydrogels can induce M2 polarization and anti‐inflammatory cytokine production (IL6 and IL10), upregulate the expression of collagen (*ACAN*, *SOX9* and *COL2*), and downregulate the expression of *MMPs* for the first time, thereby regenerating the IVD by regulating the anabolism/catabolism balance of the ECM in the NP through the IL17 signaling pathway. In addition, the hydrogel can be injected into the intervertebral space with minimal invasiveness and has a favorable HAP‐EGCG loading strategy, stabilizing the HAP‐EGCG nanorods in the intervertebral space and releasing them slowly. The injection of HAP‐EGCG@CS‐HA hydrogels led to a preferable ECM environment in vivo via the inhibition of ECM catabolism through macrophage polarization regulation and promotion of ECM anabolism, thus providing new insights for treating IVDD.

We believe that this HAP‐EGCG@CS‐HA hydrogels‐based immunomodulation can be applied in other degenerative diseases in which ECM anabolism and catabolism imbalance occur. In IVD repair, a biomaterial scaffold serves dual roles: delivering bioactive agents and mechanically supporting cells during regeneration. Due to gradual IVD regeneration, an enduring and efficacious bioactive delivery system is crucial. Concurrently, modulating the disk microenvironment shows promise in activating resident stem cells. Such an innovative approach holds promise as an avant‐garde frontier in the realm of IVD regeneration, thereby offering uncharted avenues for therapeutic intervention.

## Conflict of Interest

The authors declare no conflict of interest.

## Ethical Statement

All animal experiments in this study were performed in accordance with institutional guidelines and approved by the Laboratory Animal Centre of Qilu Hospital of Shandong University (DWLL‐2019–24). The study of human tissue was approved by the institutional review board of Qilu Hospital of Shandong University (KYLL‐2022(ZM)−923).

## Supporting information

Supporting Information

## Data Availability

The data that support the findings of this study are available from the corresponding author upon reasonable request.

## References

[1] E. J. Novais , V. A. Tran , S. N. Johnston , K. R. Darris , A. J. Roupas , G. A. Sessions , I. M. Shapiro , B. O. Diekman , M. V. Risbud , Nat. Commun. 2021, 12, 5213. 34480023 10.1038/s41467-021-25453-2 PMC8417260

[2] G. B. D. Disease , I. Injury , C. Prevalence , Lancet 2016, 388, 1545.27733282

[3] I. Urits , A. Capuco , M. Sharma , A. D. Kaye , O. Viswanath , E. M. Cornett , V. Orhurhu , Curr. Pain Headache Rep. 2019, 23, 65. 31359164 10.1007/s11916-019-0804-y

[4] B. Wu , C. Meng , H. Wang , C. Jia , Y. Zhao , Biomed. Pharmacother. 2016, 84, 754. 27716589 10.1016/j.biopha.2016.09.077

[5] J. Shen , A. Chen , Z. Cai , Z. Chen , R. Cao , Z. Liu , Y. Li , J. Hao , Bioact Mater 2022, 12, 153. 35310385 10.1016/j.bioactmat.2021.10.013 PMC8897073

[6] L. Wang , T. He , J. Liu , J. Tai , B. Wang , L. Zhang , Z. Quan , Front Immunol 2021, 12, 666355. 34122424 10.3389/fimmu.2021.666355 PMC8190407

[7] L. Smith , J. Chiaro , N. Nerurkar , D. Cortes , S. Horava , N. Hebela , R. Mauck , G. Dodge , D. Elliott , Eur. Cell Mater. 2011, 22, 291. 22102324 10.22203/ecm.v022a22 PMC3424069

[8] C. R. Harrell , B. S. Markovic , C. Fellabaum , A. Arsenijevic , V. Volarevic , Biomed. Pharmacother. 2019, 109, 2318. 30551490 10.1016/j.biopha.2018.11.099

[9] C. Manferdini , F. Paolella , E. Gabusi , Y. Silvestri , L. Gambari , L. Cattini , G. Filardo , S. Fleury‐Cappellesso , G. Lisignoli , Arthritis Res. Ther. 2016, 18, 83. 27044395 10.1186/s13075-016-0983-4 PMC4820904

[10] N. Fahy , M. L. De Vries‐Van Melle , J. Lehmann , W. Wei , N. Grotenhuis , E. Farrell , P. M. Van Der Kraan , J. M. Murphy , Y. M. Bastiaansen‐Jenniskens , G. J. V. M. Van Osch , Osteoarthr. Cartil. 2014, 22, 1167. 10.1016/j.joca.2014.05.021 24911520

[11] Y. Kokubo , K. Uchida , S. Kobayashi , T. Yayama , R. Sato , H. Nakajima , T. Takamura , E. Mwaka , N. Orwotho , A. Bangirana , H. Baba , J. Neurosurg. Spine 2008, 9, 285. 18928227 10.3171/SPI/2008/9/9/285

[12] R. Nakamichi , Y. Ito , M. Inui , N. Onizuka , T. Kayama , K. Kataoka , H. Suzuki , M. Mori , M. Inagawa , S. Ichinose , M. K. Lotz , D. Sakai , K. Masuda , T. Ozaki , H. Asahara , Nat. Commun. 2016, 7, 12503. 27527664 10.1038/ncomms12503 PMC4990710

[13] A. Sivashanmugam , R. Arun Kumar , M. Vishnu Priya , S. V. Nair , R. Jayakumar , Eur. Polym. J. 2015, 72, 543.

[14] B. P. Purcell , D. Lobb , M. B. Charati , S. M. Dorsey , R. J. Wade , K. N. Zellars , H. Doviak , S. Pettaway , C. B. Logdon , J. A. Shuman , P. D. Freels , J. H. Gorman Iii , R. C. Gorman , F. G. Spinale , J. A. Burdick , Nat. Mater. 2014, 13, 653. 24681647 10.1038/nmat3922 PMC4031269

[15] S. Bai , X. Zhang , X. Lv , M. Zhang , X. Huang , Y. Shi , C. Lu , J. Song , H. Yang , Adv. Funct. Mater. 2020, 30, 1908381.

[16] H. Zhang , H. Huang , G. Hao , Y. Zhang , H. Ding , Z. Fan , L. Sun , Adv. Funct. Mater. 2021, 31, 2006697.

[17] Y. Han , B. Jia , M. Lian , B. Sun , Q. Wu , B. Sun , Z. Qiao , K. Dai , Bioact. Mater. 2021, 6, 2173. 33511315 10.1016/j.bioactmat.2020.12.018 PMC7814104

[18] H. Yuan , X. Zheng , W. Liu , H. Zhang , J. Shao , J. Yao , C. Mao , J. Hui , D. Fan , Colloids Surf. B Biointerfaces 2020, 192, 111041. 32330818 10.1016/j.colsurfb.2020.111041

[19] N. Zhang , N. Yang , L. Zhang , B. Jiang , Y. Sun , J. Ma , K. Cheng , F. Peng , Chem. Eng. J. 2020, 402, 126200.

[20] Y. Tian , Z. Bao , Y. Ji , X. Mei , H. Yang , Drug Des. Devel. Ther. 2020, 14, 2113. 10.2147/DDDT.S251623 PMC7266312 32546974

[21] S.‐H. Kim , K. Kim , B. S. Kim , Y.‐H. An , U.‐J. Lee , S.‐H. Lee , S. L. Kim , B.‐G. Kim , N. S. Hwang , Biomaterials 2020, 242, 119905. 32145505 10.1016/j.biomaterials.2020.119905

[22] K. Ariga , Mater. Chem. Front. 2017, 1, 208.

[23] K. Ariga , T. Mori , T. Kitao , T. Uemura , Adv. Mater. 2020, 32, 1905657. 10.1002/adma.201905657 32191374

[24] K. Ariga , Trends Chem. 2020, 2, 779.

[25] K. Ariga , D. T. Leong , T. Mori , Adv. Funct. Mater. 2017, 28, 1702905.

[26] Y. Gan , J. He , J. Zhu , Z. Xu , Z. Wang , J. Yan , O. Hu , Z. Bai , L. Chen , Y. Xie , M. Jin , S. Huang , B. Liu , P. Liu , Bone Res. 2021, 9, 37. 34400611 10.1038/s41413-021-00163-z PMC8368097

[27] M. Locati , G. Curtale , A. Mantovani , Annu Rev. Pathol. 2020, 15, 123. 31530089 10.1146/annurev-pathmechdis-012418-012718 PMC7176483

[28] T. Ugai , J. P. Väyrynen , K. Haruki , N. Akimoto , M. C. Lau , R. Zhong , J. Kishikawa , S. A. Väyrynen , M. Zhao , K. Fujiyoshi , A. Dias Costa , J. Borowsky , K. Arima , J. L. Guerriero , C. S. Fuchs , X. Zhang , M. Song , M. Wang , M. Giannakis , J. A. Meyerhardt , J. A. Nowak , S. Ogino , J Natl Cancer Inst. 2022, 114, 68. 34264325 10.1093/jnci/djab142 PMC8755510

[29] Y. Yao , X.‐H. Xu , L. Jin , Front Immunol. 2019, 10, 792. 31037072 10.3389/fimmu.2019.00792 PMC6476302

[30] V. J. Devi , A. Radhika , P. G. Biju , Immunobiology 2023, 228, 152362. 36863089 10.1016/j.imbio.2023.152362

[31] X. Wang , Y. Feng , C. Chen , H. Yang , X. Yang , Lwt 2020, 131, 109810.

[32] L. Li , N. Wang , X. Jin , R. Deng , S. Nie , L. Sun , Q. Wu , Y. Wei , C. Gong , Biomaterials 2014, 35, 3903. 24507411 10.1016/j.biomaterials.2014.01.050

[33] Y. Ling , W. Zhang , P. Wang , W. Xie , W. Yang , D.‐A. Wang , C. Fan , Bioact. Mater. 2021, 6, 2914. 33718672 10.1016/j.bioactmat.2021.02.018 PMC7917462

[34] B. Balakrishnan , A. Jayakrishnan , Biomaterials 2005, 26, 3941. 15626441 10.1016/j.biomaterials.2004.10.005

[35] S. M. Richardson , G. Kalamegam , P. N. Pushparaj , C. Matta , A. Memic , A. Khademhosseini , R. Mobasheri , F. L. Poletti , J. A. Hoyland , A. Mobasheri , Methods 2016, 99, 69. 26384579 10.1016/j.ymeth.2015.09.015

[36] C. Yu , D. Li , C. Wang , K. Xia , J. Wang , X. Zhou , L. Ying , J. Shu , X. Huang , H. Xu , B. Han , Q. Chen , F. Li , J. Tang , C. Liang , N. Slater , Bioact. Mater. 2021, 6, 3568. 33842742 10.1016/j.bioactmat.2021.03.018 PMC8022109

[37] B. Ma , S. Zhang , F. Liu , J. Duan , S. Wang , J. Han , Y. Sang , X. Yu , D. Li , W. Tang , S. Ge , H. Liu , ACS Appl. Mater. Interfaces 2017, 9, 33717. 28906099 10.1021/acsami.7b13313

[38] M. Kikuchi , T. Ikoma , S. Itoh , H. N. Matsumoto , Y. Koyama , K. Takakuda , K. Shinomiya , J. Tanaka , Compos. Sci. Technol. 2004, 64, 819.

[39] M. Sarem , M. Heizmann , A. Barbero , I. Martin , V. P. Shastri , Proc. Natl. Acad. Sci. U.S.A. 2018, 115, E6135. 29915064 10.1073/pnas.1805159115 PMC6142224

[40] X. C. Li , S. J. Luo , W. Fan , T. L. Zhou , C. M. Huang , M. S. Wang , J. Orthop. Res. 2022, 40, 2488. 35170802 10.1002/jor.25292

[41] F. Cai , S. Liu , Y. Lei , S. Jin , Z. Guo , D. Zhu , X. Guo , H. Zhao , X. Niu , Y. Xi , Z. Wang , G. Chen , Cell. Immunol. 2021, 368, 104421. 34385001 10.1016/j.cellimm.2021.104421

[42] W. Li , S. Zhang , D. Wang , H. Zhang , Q. Shi , Y. Zhang , M. Wang , Z. Ding , S. Xu , B. Gao , M. Yan , Front. Cell Dev. Biol. 2021, 9, 822149. 35223870 10.3389/fcell.2021.822149 PMC8870130

[43] P. J. Roughley , Spine 2004, 29, 2691. 15564918 10.1097/01.brs.0000146101.53784.b1

[44] D. Sakai , S. Grad , Adv. Drug Deliv. Rev. 2015, 84, 159. 24993611 10.1016/j.addr.2014.06.009

[45] F. Fang , D. Jiang , Biosci. Rep. 2016, 36, e00379. 27512095 10.1042/BSR20160118 PMC5025813

[46] J. E. Mayer , J. C. Iatridis , D. Chan , S. A. Qureshi , O. Gottesman , A. C. Hecht , Spine J. 2013, 13, 299. 23537453 10.1016/j.spinee.2013.01.041 PMC3655694

[47] H. Hebelka , K. Lagerstrand , H. Brisby , P. J. Owen , M. J. Quittner , T. Rantalainen , D. L. Belavy , Eur. Spine J. 2019, 28, 2153. 31309335 10.1007/s00586-019-06059-1

[48] B. Han , K. Zhu , F.‐C. Li , Y.‐X. Xiao , J. Feng , Z.‐L. Shi , M. Lin , J. Wang , Q.‐X. Chen , Spine 2008, 33, 1925. 18708924 10.1097/BRS.0b013e31817c64a9

[49] Y. Jin , R. H. Koh , S.‐H. Kim , K. M. Kim , G. K. Park , N. S. Hwang , Mater. Sci. Eng., C 2020, 115, 111096. 10.1016/j.msec.2020.111096 32600700

[50] D.‐W. Zhao , C. Liu , K.‐Q. Zuo , P. Su , L.‐B. Li , G.‐Y. Xiao , L. Cheng , Chem. Eng. J. 2021, 408, 127362.

[51] J. Montero , V. Gómez‐Abellán , M. Arizcun , V. Mulero , M. P. Sepulcre , Fish Shellfish Immunol. 2016, 55, 632. 27368534 10.1016/j.fsi.2016.06.044

[52] Z. Luo , W. Peng , Y. Xu , Y. Xie , Y. Liu , H. Lu , Y. Cao , J. Hu , Acta Biomater. 2021, 136, 519. 34551329 10.1016/j.actbio.2021.09.026

[53] L.‐L. Li , B. Dai , Y.‐H. Sun , T.‐T. Zhang , Ann. Transl. Med. 2020, 8, 674. 32617294 10.21037/atm-19-1739 PMC7327349

[54] L. Ni , Y. Zheng , T. Gong , C. Xiu , K. Li , Saijilafu , B. Li , H. Yang , J. Chen , J. Cell. Physiol. 2019, 234, 5362. 30367477 10.1002/jcp.27507

