# Supplementary material for: Decoding Macrophage Subtypes to Engineer Modulating Hydrogels for the Alleviation of Intervertebral Disk Degeneration
Source: Adv Sci (Weinh). 2023 Nov 8;11(1):2304480. doi: 10.1002/advs.202304480 (PMC10767410; doi:10.1002/advs.202304480)
Supplement: Supplementary file 1 — Supporting Information [file ADVS-11-2304480-s001.pdf]

ADVANCED SCIENCE
Open Access

Supporting Information

for *Adv. Sci.*, DOI 10.1002/advs.202304480

Decoding Macrophage Subtypes to Engineer Modulating Hydrogels for the Alleviation of Intervertebral Disk Degeneration

Da-Wang Zhao, Qian Cheng, Huimin Geng, Jinbo Liu, Yuanqiang Zhang, Jiwei Cui, Chao Liu* and Lei Cheng**

Supporting Information

Title name

Decoding macrophage subtypes to engineer modulating hydrogels for the alleviation of intervertebral disk degeneration

Author information

Da-Wang Zhao[1], Qian Cheng[1], Huimin Geng, Jinbo Liu, Yuanqiang Zhang, Jiwei Cui, Chao Liu*, Lei Cheng**

Experimental Section

Materials

Oleic acid, $CaCl_2$ (99.0%), $NaH_2PO_4{\cdot}2H_2O$ (98%) were purchased from Sinopharm (Shanghai, China), EGCG (＞95%) was obtained from meilunbio (China), CS (MW 20-30 kDa, degree of carboxylation ~80%) was purchased from Beijing Solarbio Science & Technology Co., Ltd. (China). Sodium hyaluronate (200-400 k) was obtained from Bloomage Freda Biopharm Co., Ltd. (China). Sodium periodate ($NaIO_4$) was purchased from Damao Chemical Reagent Co., Ltd (Tianjin, China), 1,1-diphenyl-2-picrylhydrazyl (DPPH, 98%) was obtained from Yuanye Biology (China), Rhodamine B and methylene blue were purchased from Fuchen Chemical Reagent Co., Ltd. (Tianjin, China). Phosphate buffered saline (PBS), were purchased from Biological Industries Science &Technology Co. Ltd. (China).

Mouse anti-MMP2 (66366-1-Ig, Proteintech, China) was obtained from Proteintech, China. Other reagents were mouse anti-iNOS (ab49999, Abcam, USA), rabbit anti-mannose receptor (ab64693, Abcam, USA), mouse anti-aggrecan (ACAN, NB600-504, Novus, USA), APC anti-rat CD11b/c (Biolegend, USA), PE anti-rat CD86 (Biolegend, USA), mouse anti-rat CD163 (Bio-Rad, USA), DyLight 488, goat anti-mouse IgG (Abbkine, USA), DyLight 594, goat anti-rabit IgG (Abbkine, USA), M-CSF (Peprotech, USA), phalloidine (YEASEN, China), and DAPI (Beyotime, China).

Methods

Fabrication and characterization of HAP-EGCG

HAP nanorods were first prepared according to the previous report.[1] The resulting product was dried into powder for later use. Second, 12 mg of EGCG was dissolved in 6 mL of tris-HCl (10 mM, pH=8.5) with a concentration of 2 mg/mL. 10 mg of HAP was added in the EGCG solution, dispersed by ultrasound and shaken at room temperature for 24 h to form the HAP-EGCG complex. After being centrifuged and washed with water twice (3000 rcf, 10 mins), the HAP-EGCG nanorods were dispersed in deionized water for characterization, in PBS solution for biological experiments. To confirm the decoration of EGCG on the HAP surfaces, Fourier transform infrared (FTIR) spectra were monitored on a FTIR spectrometer (Bruker Tensor II, Germany) in the range of 500-4000 cm^{-1}. UV-vis spectra were recorded on a Shimadzu UV-2600 (Japan) spectrophotometer. The zeta potential was measured using a Malvern Zetasizer (Nano ZS90, UK). The morphology and elemental analysis were imaged by scanning electron microscopy (SEM, Zeiss G300, Germany). The radical scavenging activity of HAP-EGCG was determined by the 2,2-diphenyl-1-picrylhydrazyl (DPPH). 0.05 mg/mL ~ 1 mg/mL of HAP-EGCG was incubated with 1mL of DPPH (0.2 mM in ethanol) for 30 mins at room temperature (n=3). The DPPH radical scavenging activity was determined by UV-vis spectra at 517 nm, which was calculated following the equation: [control optical density (OD) - sample OD] ×100/ control OD.

Synthesis and characterization of HA and CS

HA was prepared according to previous report.[2] The actual oxidation degree of HA was calculated to be 25% in this work according to the corresponding reaction process and calculation formula given in the previous literature.[3] The molecular structures of HA and CS were characterized by proton nuclear magnetic resonance (^{1}H-NMR) spectra.

Preparation and characterization of HAP-EGCG@CS-HA hydrogels

CS and HA were respectively dissolved in deionized water at desired concentrations overnight in the 4 ^{o}C refrigerator. The pH of both solutions was adjusted to 7.4, and HAP-EGCG nanorods was added into the solution of HA. The concentration of CS was 40 mg/mL and the concentration of HA was 30 mg/mL containing 1 mg/mL HAP-EGCG nanorods. Then, the CS-HA hydrogels was prepared by mixing the two solutions rapidly in a volume ratio of 1: 1. The Schiff base formed from the amino group of CS and the aldehyde group of HA was analyzed by FTIR spectra. The morphology of CS-HA hydrogels and HAP-EGCG nanorods were measured by SEM. Before observation, the freeze-dried hydrogels were sputtered with platinum for 60 s at 10 mA. The porosity of hydrogels was mesured by ethanol displacement method with reference to previous work[4]. Briefly, the prepared hydrogels were freeze-dried and their weights and volumes were recorded, followed by complete submergence into ethanol until saturation. The percentage of the porosity was calculated as following equation:

Porosity (%) = (W_s - W_d) / (V_s × ρ *ethanol)*

Where, W_s is the weight of the drowned hydrogels after submergence into ethanol, W_d is the weight of the freeze-dried hydrogels, V_s is the volume of the freeze-dried hydrogels and ρ is the density of ethanol.

Evaluation of the injectability, self-healing and biodegradable nature of HAP-EGCG@CS-HA hydrogels

Equivalent volumes of CS and HA stained with red, blue and purple were loaded into separate syringes with a “Y” shape connector. The mixed solutions were injected through a narrow needle (26 G) onto a plastic plate, forming different patterns of hydrogels at room temperature. The obtained hydrogels were sliced into two halves, and the three pieces of hydrogels with different colors were combined together. A small amount of PBS solution was added, the three pieces of hydrogels healed into one which could be suspended under gravity after 3 mins. The above properties were evaluated using a digital camera. The mechanical properties of hydrogels were quantitatively characterized by RS6000 rheometer (HAAKE) using a cone-plate system (C35/1° Ti L07116, diameter 35 mm, and core angle 1°) at 37 ^{o}C. The storage modulus (G′) and loss modulus (G″) were recorded as functions of time at a frequency sweep of 1 Hz and a strain level of 1%. The gelation time (t_{gel}) was defined as the time when G′ became higher than G″. The stress-strain test was established by applying a low strain (γ = 0.1%) to the hydrogel for the first 100 s, then a high strain (γ = 1000%) for the next 50 s, and repeated twice. To study the biodegradability of

the hydrogel, three groups of 400 μL of hydrogels were incubated in PBS solution at 37 °C, which were weighted on days 2, 4, 6, 9, 12 (W_t). The biodegradation level was determined by the equation W_t/W_0.

Preparation of the leach liquor

Three groups of hydrogels (the hydrogel without nanorods (CS-HA), the hydrogel containing HAP nanorods (HAP@CS-HA), and the hydrogel containing HAP-EGCG (HAP-EGCG@CS-HA) were obtained within 10 minutes. 1 mL of hydrogel was added to 10 mL of DMEM culture medium and incubated at 37 °C for 24 h. The leach liquor was then filtered with a disposable sterile filter (0.22 μm) for later use.

The isolation and culture of rBMMSC and macrophage

It has been confirmed that the existence of stem cells in intervertebral disk (IVD), however, the isolation of stem cells in IVD is complicated, and the small quantity limits the use for further experiments.[5] Therefore, we use rBMMSCs to explore the ability of HAP-EGCG@CS-HA hydrogels in inducing nucleopulpogenic differentiation *in vitro*. The rBMMSCs were isolated from 4-week-old Sprague Dawley rats. Firstly, the bilateral femurs of rats were taken out, both ends of femurs were cut off by scissors, the cells were flushed from the middle femoral marrow cavity by DMEM. The rBMMSCs were cultured in DMEM (Gibco, USA) containing 10% fetal bovine serum (FBS, Gibco, USA), 100 U/mL penicillin and 100 μg/mL streptomycin (Gibco, USA) in incubator with the atmosphere of 37 °C, 95% O2 and

5% CO2. The culture medium was changed every 2 days. The isolation, culture and identifications of macrophages were carried out according to previously described methods.[6, 7] In summary, we isolated cells from the bone marrow cavity of rats. Subsequently, these cells were subjected to M-CSF stimulation and cultured in a high-glucose DMEM medium. After a duration of 5 days, primary macrophage cells were successfully derived.

Immunofluorescence (IF) staining

After culturing for desired time, rBMMSCs (2 days) or macrophages (3 days) for IF staining were fixed with 4% paraformaldehyde. Cells were then washed with pre-cooling PBS and subsequently blocked in 0.1% Triton X-100 for 10 mins. Then, the cells were fixed and incubated with primary antibodies at 1:100 dilution overnight at 4 ^{o}C. Then, the cells were incubated with secondary antibodies at 1:500 dilution for one hour. Furthermore, DAPI (Beyotime, China) and phalloidin (YEASEN, China) were used to stain the nuclei and actin. Finally, the samples were observed under confocal mode using a confocal microscopy (Opera Phenix, PerkinElmer, USA). We also used ImageJ software to quantify the expression of target protein.

Methyl Thiazolyl Tetrazolium (MTT) assay

rBMMSCs (6×10^4/mL) or macrophages (6×10^4/mL) were cultured in leach liquor in 24-well plates for 0, 2, 4, 6, and 8 days and exposed to fresh media every other day. The control group was cultured in a regular cell culture medium. During the last 4

hours of each timepoints of culture, the culture medium was discarded and the cells were cultured with MTT solution and DMEM. Finally, after discarding all the solution, formazan solution was added, and detected at 490 nm to identify cell percent viability.

Live/Dead Cell staining

40 μL of rBMMSCs (6×10^7/mL) or macrophages (6×10^7/mL) cell suspension was cocultured with 400 μL of different hydrogels (CS-HA, HAP@CS-HA, HAP-EGCG@CS-HA) for 1 day. Then the cells in hydrogels was stained with 500 μL of Calcein-AM/PI kit (Solarbio, China) according manufactory's instructions. Then the live/dead cells in hydrogels was observed with confocal microscopy (Opera Phenix, PerkinElmer, USA). The number of live or dead cells was quantified in triplicate using High Content Screening System.

Cell apoptosis

The rBMMSCs (6×10^4/mL) or macrophages (6×10^4/mL) were harvested after culturing in leach liquor for 1 day. Then, cells were stained with 7-AAD/Annexin V (detects apoptotic cells) and detected using a Gallios flow cytometer (Beckman, USA).

Flow cytometry and Enzyme-linked immunosorbent assay (ELISA)

After culturing in 6-well plate for 3 days, the cells were scratched into centrifuge and stained with fluorescently-labeled antibodies according to the previous study [6]. After

culturing macrophages (6×10^4/mL) in leach liquor for 3 days, the expressions of cytokines (TNFα, IL4, IL6, and IL10) of were detected in triplicate by ELISA kit according manufactory's instructions.

Reverse transcription-polymerase chain reaction (RT-PCR)

The cells were culturing in leach liquor in the 6-well plate for the desired time, then, the total mRNA was extracted to synthesize complementary DNA (cDNA). Finally, RT-PCR was performed on a 7900HT Fast Real-Time PCR System (Applied Biosystems, USA) according to the previous study [6]. *β-actin* was used as housekeeping gene. The primers were shown in Table S1.

Table S1. Primers used in the RT-PCR.

Gene	Primers Sequence (F, forward; R, reverse; 5'−3')	Product Size (bp)
ACAN	F: CTGAATGGGAGCCAGCCTAC<br>R: GATGTGGAAGGGACTTGCGA	78
SOX9	F: CACAAGAAAGACCACCCCGA<br>R: ATGTGAGTCTGTTCGGTGGC	209
COL2	F: TTTGACGAGAAGGCTGGTGG<br>R: GGACCAATGGGACCAGAGAC	164
MMP2	F: TGGCACCACCGAGGATTATG<br>R: TGCCCAGAAAAGTGAAGGGG	85
MMP13	F: ACCCAGCCCTATCCCTTGAT<br>R: TCTCGGGATGGATGCTCGTA	178
CCR7	F: CATTTTCCAGGTGTGCTTCTGC<br>R: CACCGACTCATACAGGGTGT	91
CD206	F: GAGGACTGCGTGGTGATGAA<br>R: CATGCCGTTTCCAGCCTTTC	154
BMP2	F: GCATGTTTGGCCTGAAGCAG<br>R: CGATGGCTTCTTCGTGATGG	180
β-actin	F: CCTCTATGACAACACAGT<br>R: AGCCACCAATCCACACAG	155

Macrophage conditioned culture system

The intervertebral disk degeneration (IVDD) recruits M1 infiltration and thus produces inflammatory factors which interrupt the repair of NP. Therefore, in order to verify the repair ability of the immune microenvironment produced by hydrogels induced macrophages, the conditioned culture medium was performed in our study. The macrophages (6×10^4/mL) were cultured in leach liquor in 24-well plates for 3 days, then, the mRNA of macrophages was extracted for the RT-PCR experiment, the medium was obtained and used for further rBMMSCs (6×10^4/mL) incubation. During establishment of the present culture system, we chose a 1:1 ratio of rBMMSCs and macrophages to avoid disruption from cell numbers. Several papers support our strategy, and a 1:1 ratio of stem cells and macrophages has been accepted by most studies.[8] The rBMMSCs were incubated for the desired time and were performed with IF and RT-PCR in triplicate to identify the anti-inflammatory and nucleopulpogenic ability of hydrogels.

In vivo rat IVDD model

The animal experiment was done in Qilu Hospital of Shandong University with the approval of the Ethics Committee on animal experiments of Qilu Hospital of Shandong University. Rats (Sprague Dawley, 4-week-old male, body weight 90~140g) were housed 2 per cage and kept in an environment of constant temperature and humidity. Firstly, inhalation of isoflurane into the respiratory tract of rats as anesthesia. Then, 18G needles were punctured into the center of IVD (C3-4, C4-5,

C5-6, C6-7) under X-ray after preoperative disinfection. The needle rotates 360 degrees in the IVD and stays for 30 s before being pulled out. Then, 0.04 mL of the hydrogels (CS-HA, HAP@CS-HA, HAP-EGCG@CS-HA) was injected into the puncture site of the IVD by a 1 mL syringe. C7-8 was used as control group and no puncture or injection was performed in this section. All operations were strictly complied with sterility requirements and performed as described elsewhere in triplicate.[9]

Magnetic resonance imaging (MRI)

MRI experiments were performed on rats at 0 (before surgery), 4, and 8 weeks (after surgery) on a 3.0T MRI scanner (GE, USA). Six slices with 3.0 mm slice thickness were measured per scan with parameter for sequence T2W1: TR= 3000 ms, TE1 = 68.0 ms, reconstruction matrix: 512×512, slice thickness = 3.0 mm. The gray value of IVD was identified using ImageJ 1.52 k software.

Tissue section staining

IVD samples (n=3) were fixed and embedded in paraffin. The thick of 6 μm slides were sectioned and stained with hematoxylin and eosin (HE) and Safranin O. The stained slides were covered and observed with microscope (Leica, DM4 B, Germany) to examine the histopathological changes.

RNA sequencing

The number of differentially expressed genes in HAP-EGCG@CS-HA hydrogels and Control groups, the fold change of macrophage polarization-related genes, the Kyoto Encyclopedia of Genes and Genomes (KEGG) enrichment of up/down-regulated pathways, and the Gene Ontology (GO) enrichment of cell function were examined by RNA sequencing (LC, China).

Statistical analysis

Data are shown as the mean ± standard deviation (SD) from at least three independent experiments. The normality of data was first analyzed. For data conforming normal distribution, statistical differences among groups were analyzed by SPSS Statistics 23 using one-way ANOVA with a post-hoc bonferroni test. *P < 0.05 compared with Control. #P < 0.05 compared with CS-HA hydrogels. △P < 0.05 compared with HAP@CS-HA hydrogels. \$$P$ < 0.05 compared with HAP-EGCG@CS-HA hydrogels. **P < 0.01 compared with Control. ##P < 0.01 compared with CS-HA hydrogels. △△P < 0.01 compared with HAP@CS-HA hydrogels. \$\$$P$ < 0.01 compared with HAP-EGCG@CS-HA hydrogels. Statistic differences were considered significantly at P < 0.05.

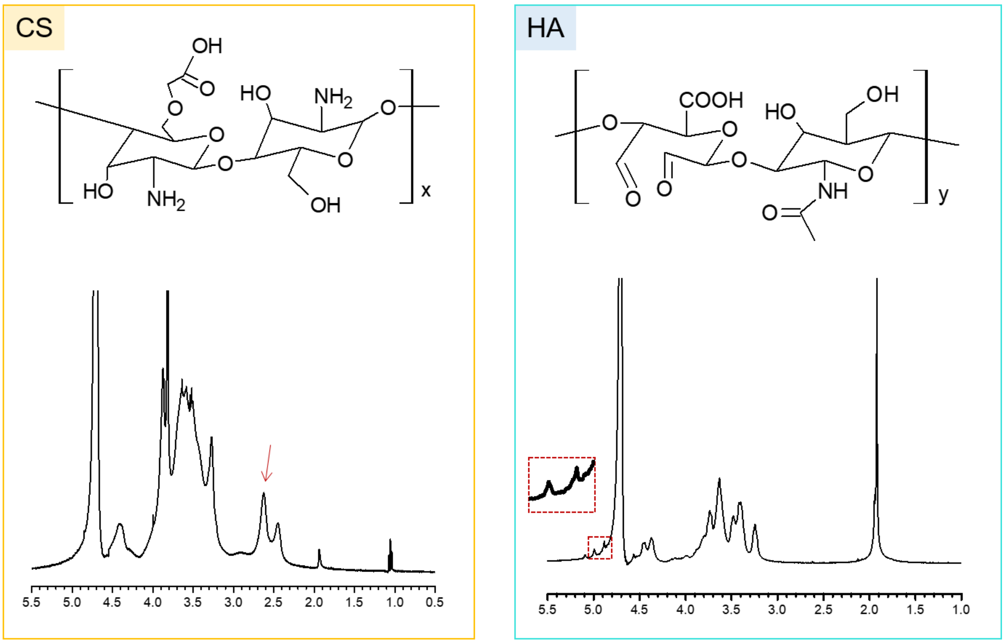

Figure S1. ^{1}H-NMR spectra of CS and HA.

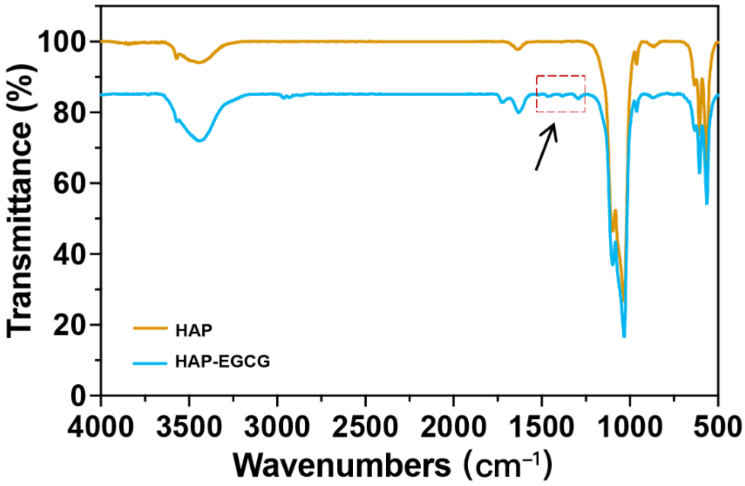

Figure S2. FTIR spectra of HAP and HAP-EGCG.

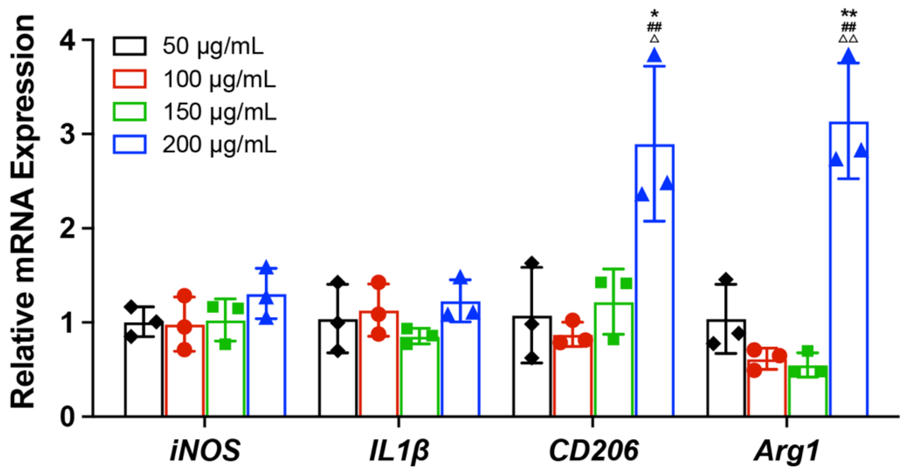

Figure S3. RT-PCR results for *iNOS*, *IL1β*, *CD206*, and *Arg1* mRNA of macrophages cultured in leaching liquors of the hydrogels on day 3.
(n = 3. The values presented are the mean±SD. Statistical significance was assessed by one-way ANOVA with a post-hoc bonferroni test. *, #, and △ represent $P < 0.05$ compared with 50 μg/mL, 100 μg/mL, and 150 μg/mL, respectively; **, ##, and △△ represent $P < 0.01$ compared with 50 μg/mL, 100 μg/mL, and 150 μg/mL, respectively)

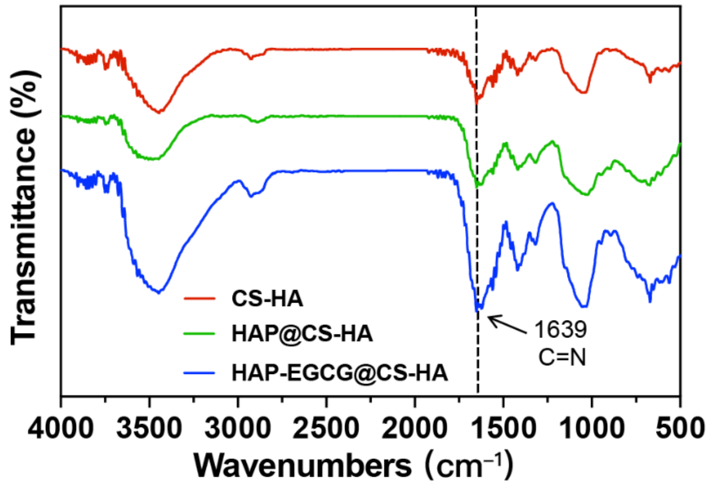

Figure S4. FTIR spectra of CS-HA hydrogels, HAP@CS-HA hydrogels and HAP-EGCG@CS-HA hydrogels.

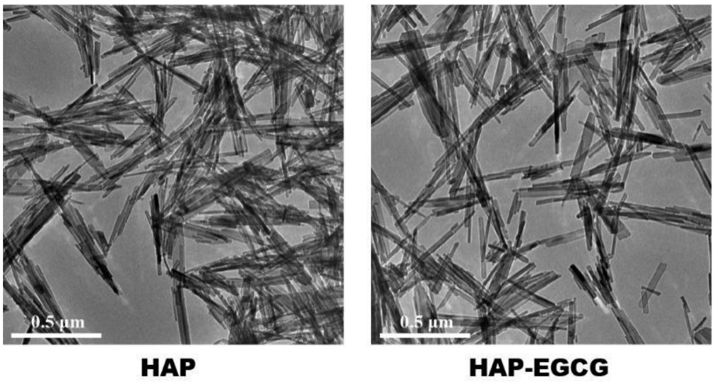

Figure S5. TEM images of HAP and HAP-EGCG.

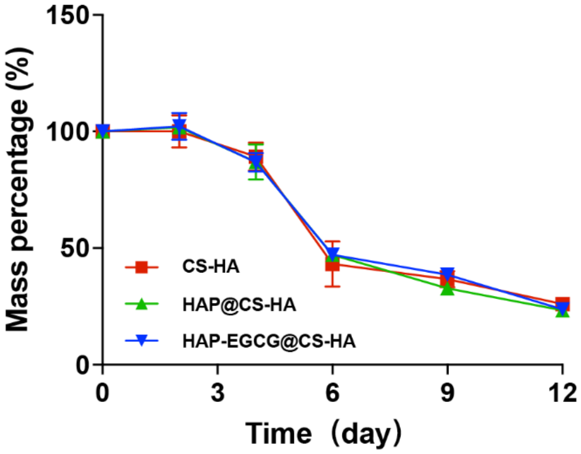

Figure S6. The degradable properties of hydrogels *in vitro* (CS-HA, HAP@CS-HA, HAP-EGCG@CS-HA).

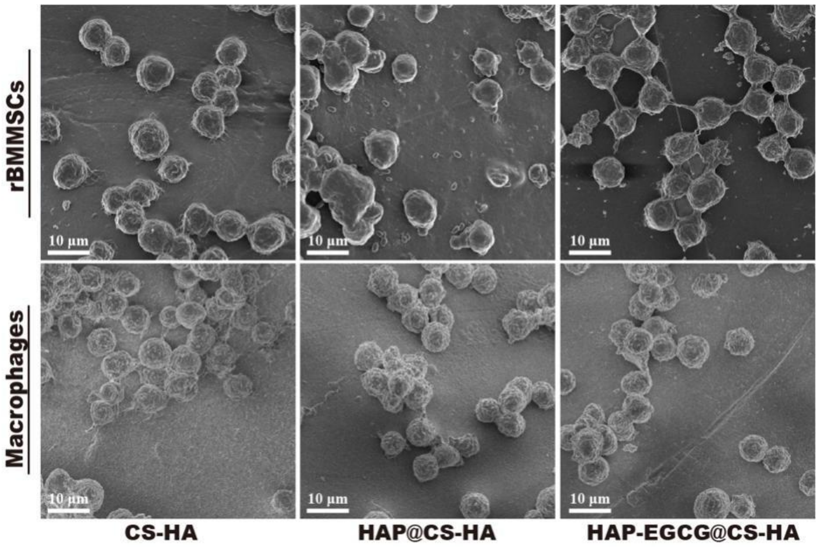

Figure S7. Morphology and adhesion of rBMMSCs and macrophages on samples as imaged by SEM on day 2.

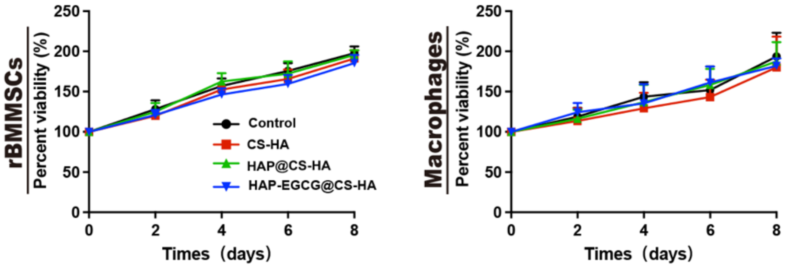

Figure S8. Percent viability of rBMMSCs and macrophages cultured in leach liquor for 2, 4, 6, and 8 days by MTT test.

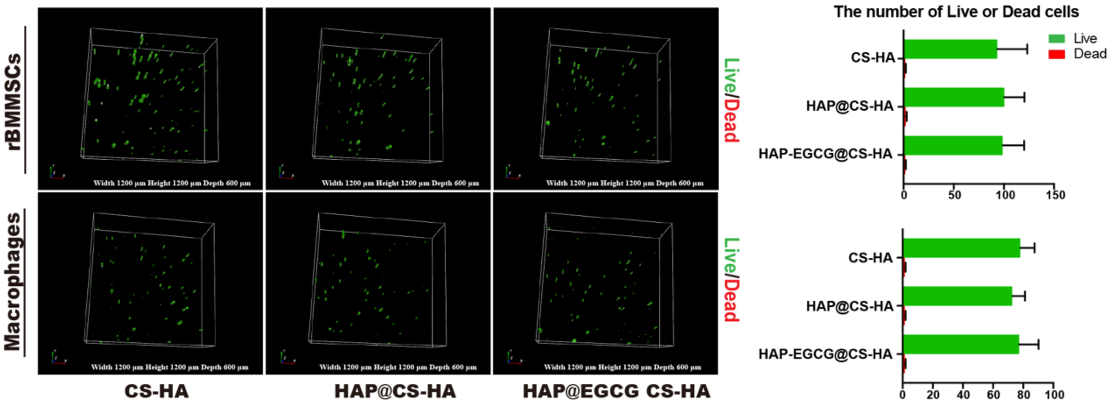

Figure S9. Live/dead staining images and quantitative analysis of the viability of rBMMSCs and macrophages on day 1.

(n = 3. The values presented are the mean±SD. Statistical significance was assessed by one-way ANOVA with a post-hoc bonferroni test. # and △ represent $P < 0.05$ compared with CS-HA hydrogels and HAP@CS-HA hydrogels, respectively; ## and △△ represent $P < 0.01$ compared with CS-HA hydrogels and HAP@CS-HA hydrogels, respectively)

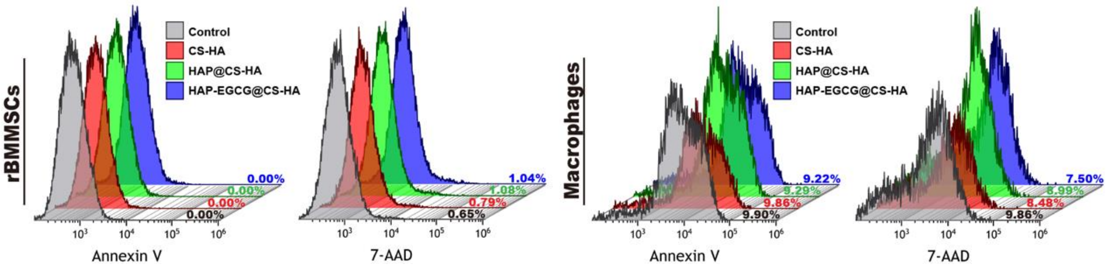

Figure S10. Percentages of apoptotic rBMMSCs and macrophages cultured on samples on day 1 using Annexin V/7-AAD staining determined by flow cytometry.

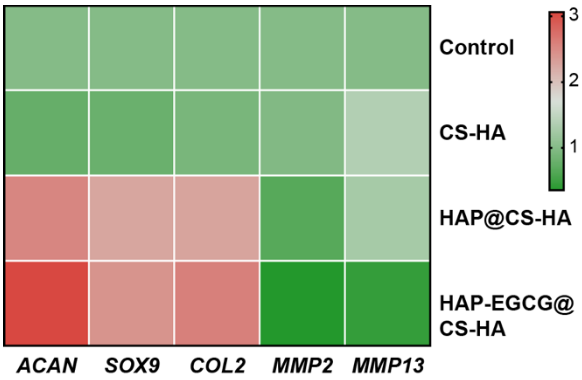

Figure S11. RT-PCR results for *ACAN*, *SOX9*, *COL2*, *MMP2*, and *MMP13* mRNA of rBMMSCs cultured in leaching liquors of the hydrogels on day 2.

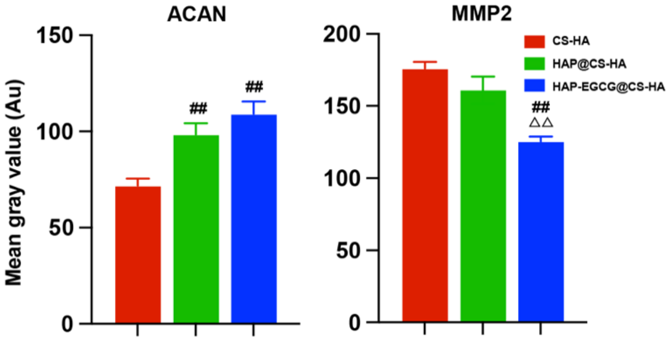

Figure S12. Quantitative analysis of ACAN and MMP2 of rBMMSCs in hydrogels on day 2 by IF staining.

(n = 3. The values presented are the mean±SD. Statistical significance was assessed by one-way ANOVA with a post-hoc bonferroni test. # and △ represent $P < 0.05$ compared with CS-HA hydrogels and HAP@CS-HA hydrogels, respectively; ## and △△ represent $P < 0.01$ compared with CS-HA hydrogels and HAP@CS-HA hydrogels, respectively)

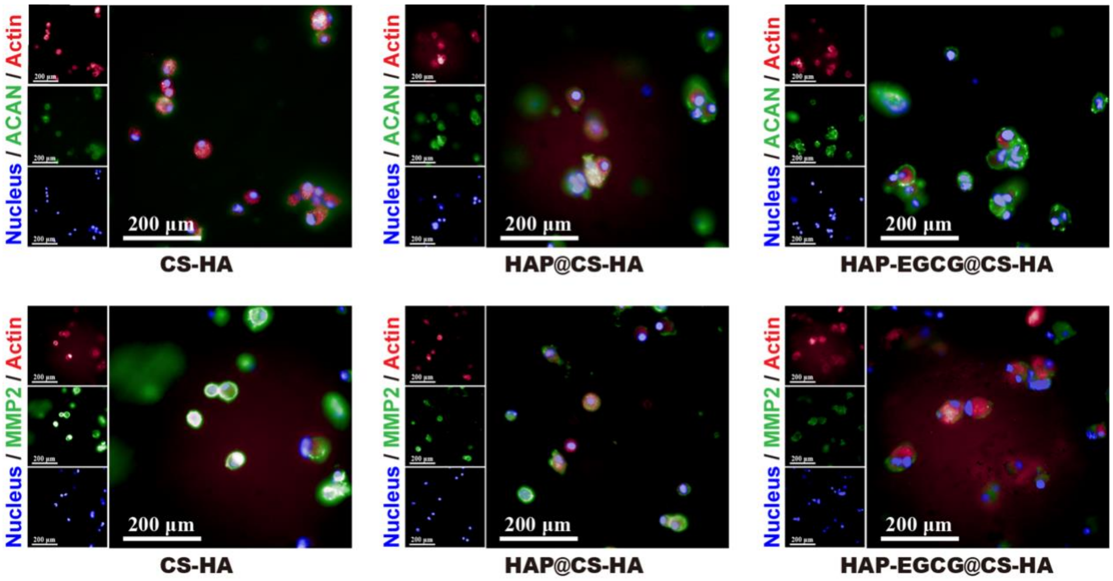

Figure S13. Expression of ACAN and MMP2 of rBMMSCs in hydrogels on day 2 by IF staining.

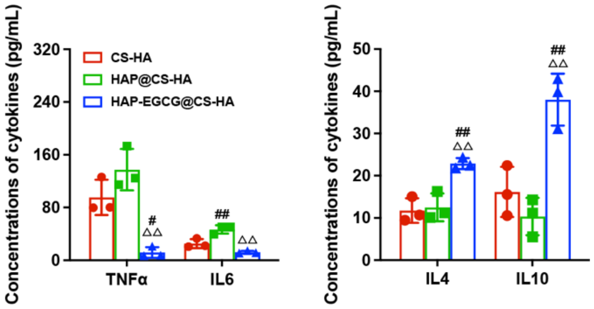

Figure S14. TNFα, IL6, IL4, and IL10 production by macrophages cultured on hydrogels on day 3 by ELISA.

(n = 3. The values presented are the mean±SD. Statistical significance was assessed by one-way ANOVA with a post-hoc bonferroni test. # and △ represent *P* < 0.05 compared with CS-HA hydrogels and HAP@CS-HA hydrogels, respectively; ## and △△ represent *P* < 0.01 compared with CS-HA hydrogels and HAP@CS-HA hydrogels, respectively)

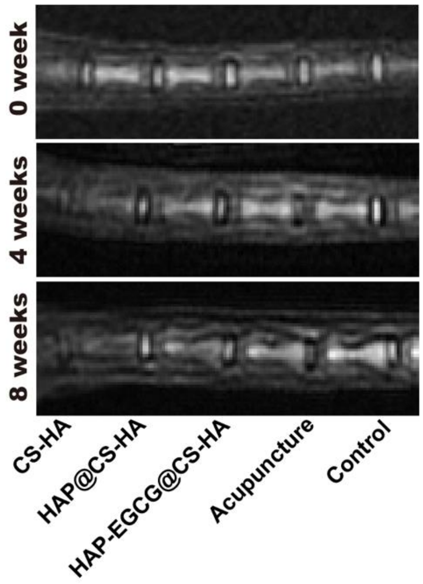

Figure S15. Coronal images from T2 MRI at 0 (normal group), 4, and 8 weeks after injection. Notably, the highest water content and the lowest histological grade were found in the HAP-EGCG@CS-HA hydrogels group, suggesting that HAP-EGCG@CS-HA hydrogels had the best ability to regulate the ECM anabolism/catabolism balance *in vivo*.

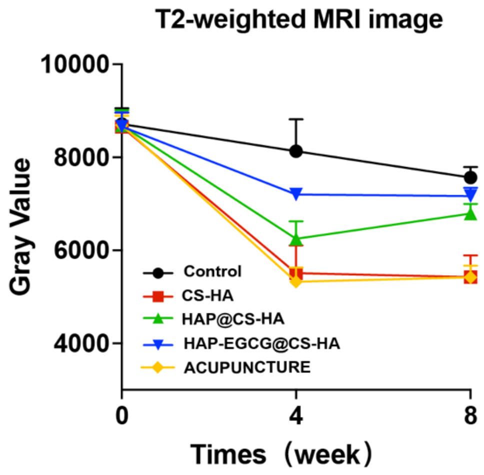

Figure S16. Gray value of the IVD in T2 MRI images at 0, 4, and 8 weeks after injection. (n = 3. The values presented are the mean±SD)

References

1. Ma, B.; Zhang, S.; Liu, F.; Duan, J.; Wang, S.; Han, J.; Sang, Y.; Yu, X.; Li, D.; Tang, W.; Ge, S.; Liu, H. *ACS Appl Mater Interfaces* **2017,** 9, 33717-33727.
2. Li, L.; Wang, N.; Jin, X.; Deng, R.; Nie, S.; Sun, L.; Wu, Q.; Wei, Y.; Gong, C. *Biomaterials* **2014,** 35, 3903-17.
3. Ma, X.; Xu, T.; Chen, W.; Qin, H.; Chi, B.; Ye, Z. *Carbohydr Polym* **2018,** 179, 100-109.
4. Ahmadian, Z.; Correia, A.; Hasany, M.; Figueiredo, P.; Dobakhti, F.; Eskandari, M. R.; Hosseini, S. H.; Abiri, R.; Khorshid, S.; Hirvonen, J.; Santos, H. A.; Shahbazi, M. A. *Adv Healthc Mater* **2021,** 10, e2001122.
5. Wangler, S.; Peroglio, M.; Menzel, U.; Benneker, L. M.; Haglund, L.; Sakai, D.; Alini, M.; Grad, S. *Spine (Phila Pa 1976)* **2019,** 44, 1613-1622.
6. Zhao, D. W.; Liu, C.; Zuo, K. Q.; Su, P.; Li, L. B.; Xiao, G. Y.; Cheng, L. *Chem Eng J* **2021,** 408.
7. Zhao, D. W.; Ren, B.; Wang, H. W.; Zhang, X.; Yu, M. Z.; Cheng, L.; Sang, Y. H.; Cao, S. S.; Thieringer, F. M.; Zhang, D.; Wan, Y.; Liu, C. *Bone Joint Res* **2021,** 10, 411-424.
8. Zhao, D. W.; Du, C. M.; Zuo, K. Q.; Zhao, Y. X.; Xu, X. Q.; Li, Y. B.; Tian, S.; Yang, H. R.; Lu, Y. P.; Cheng, L.; Xiao, G. Y. *Adv Healthc Mater* **2023,** 12, e2202537.
9. Chen, W.; Chen, H.; Zheng, D.; Zhang, H.; Deng, L.; Cui, W.; Zhang, Y.; Santos, H. A.; Shen, H. *Adv Sci (Weinh)* **2020,** 7, 1902099.
